# Triterpenes and Phenolic Compounds from the Fungus *Fuscoporia torulosa*: Isolation, Structure Determination and Biological Activity

**DOI:** 10.3390/molecules26061657

**Published:** 2021-03-16

**Authors:** Zoltán Béni, Miklós Dékány, András Sárközy, Annamária Kincses, Gabriella Spengler, Viktor Papp, Judit Hohmann, Attila Ványolós

**Affiliations:** 1 Spectroscopic Research, Gedeon Richter Plc., Gyömrői út 19-21, H-1103 Budapest, Hungary; z.beni@richter.hu (Z.B.); M.Dekany@richter.hu (M.D.); 2 Department of Pharmacognosy, University of Szeged, Eötvös u. 6, H-6720 Szeged, Hungary; sarkozy@pharmacognosy.hu; 3 Department of Medical Microbiology and Immunobiology, University of Szeged, Dóm Square 10, H-6720 Szeged, Hungary; kincses.annamaria@med.u-szeged.hu (A.K.); spengler.gabriella@med.u-szeged.hu (G.S.); 4 Department of Botany, Hungarian University of Agriculture and Life Sciences, Villányi út 29-43, H-1118 Budapest, Hungary; papp.viktor@uni-mate.hu; 5 Interdisciplinary Centre for Natural Products, University of Szeged, Eötvös u. 6, H-6720 Szeged, Hungary; 6 Department of Pharmacognosy, Semmelweis University, Üllői u. 26, H-1085 Budapest, Hungary

**Keywords:** *Fuscoporia torulosa*, triterpenes, cytotoxic, antioxidant, synergism, antibacterial, ORAC, DPPH

## Abstract

Investigation of the methanol extract of the poroid fungus *Fuscoporia torulosa* resulted in the isolation of a novel triterpene, fuscoporic acid (**1**), together with inoscavin A and its previously undescribed *Z* isomer (**2** and **3**), 3,4-dihydroxy-benzaldehide (**4**), osmundacetone (**5**), senexdiolic acid (**6**), natalic acid (**7**), and ergosta-7,22-diene-3-one (**8**). The structures of fungal compounds were determined on the basis of NMR and MS spectroscopic analyses, as well as molecular modeling studies. Compounds **1**, **6**–**8** were examined for their antibacterial properties on resistant clinical isolates, and cytotoxic activity on human colon adenocarcinoma cell lines. Compound **8** was effective against Colo 205 (IC_50_ 11.65 ± 1.67 µM), Colo 320 (IC_50_ 8.43 ± 1.1 µM) and MRC-5 (IC_50_ 7.92 ± 1.42 µM) cell lines. Potentially synergistic relationship was investigated between **8** and doxorubicin, which revealed a synergism between the examined compounds with a combination index (CI) at the 50% growth inhibition dose (ED_50_) of 0.521 ± 0.15. Several compounds (**1** and **6**–**8**) were tested for P-glycoprotein modulatory effect in Colo 320 resistant cancer cells, but none of the compounds proved to be effective in this assay. Fungal metabolites **2**–**5** were evaluated for their antioxidant activity using the oxygen radical absorbance capacity (ORAC) and DPPH assays. Compounds **4** and **5** were found to have a considerable antioxidant effect with EC_50_ 0.25 ± 0.01 (DPPH) and 12.20 ± 0.92 mmol TE/g (ORAC)**.** The current article provides valuable information on both the chemical and pharmacological profiles of *Fuscoporia torulosa*, paving the way for future studies with this species.

## 1. Introduction

The members of Hymenochaetaceae family (*Phellinus* s. lato) are considered to be an abundant source of diverse bioactive metabolites, and the pharmacological potential of wild species is intensely studied around the world [1,2,3,4]. The morphologically circumscribed *Phellinus* s. lato represents a phylogenetically polyphyletic assemblage that includes smaller and more natural genera [5]. Among these, *Fuscoporia* Murrill is one of the largest genera, distributed on all forested continents [6]. On the basis of recent taxonomic studies, the genus is made up of 50 species, which are divided into six phylogenetic lineages [7,8]. The so-called “*Fuscoporia torulosa* group” comprises 10 species, of which only two have been reported in Europe: *Fuscoporia wahlbergii* (Fr.) T. Wagner & M. Fisch. and *F. torulosa* (Pers.) T. Wagner & M. Fisch. [7]. The more common *F. torulosa* is widespread in Europe with a preference for southern areas, growing on various broad-leaved trees and occasionally on conifers [9]. The perennial woody basidiocarps of *F. torulosa* mainly develop on the base of living trees and are easily distinguished by their rusty-brown color with greenish sterile surfaces due to the presence of mosses and algae [10].

The first study to explore the chemistry of *F. torulosa* appeared in the mid 1990s, which reported the isolation of nine compounds, among which were three novel lanosteroids, namely, albertic, natalic, and torulosic acids [11]. One and a half decades after the first study, Deveci et al. presented the results of an investigation on *F. torulosa* samples of Turkish origin: one new and nine known compounds were isolated. The fungal metabolites were examined for their potential cytotoxic, antioxidant, cholinesterase, and tyrosinase inhibitory activities [12]. Besides these mycochemical studies, several articles reported the antimicrobial, antioxidant, cytotoxic, and xanthine oxidase inhibitory capacities, and the antiacne activities and the phenolic profile of *F. torulosa* samples prepared with solvents of different polarities [2,13,14,15].

The present study was performed with the aim of mapping the chemical profile and pharmacological potential of *F. torulosa,* thus providing more information about the fungal metabolites responsible for these biological activities. 

## 2. Results and Discussion

**Figure 1:**
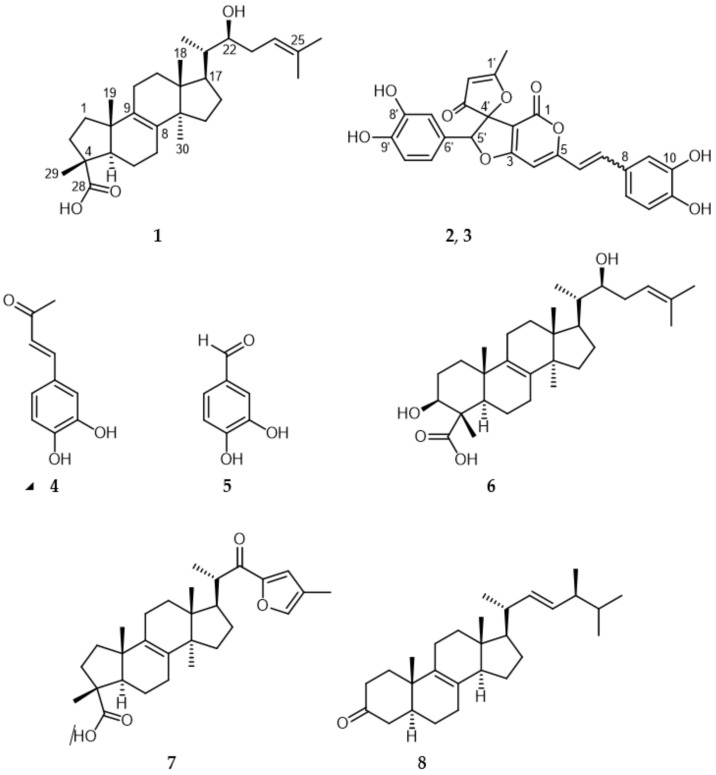
Compounds isolated from *Fuscoporia torulosa* (**2** and **3** *E*-*Z* isomers).

An in-depth chemical examination of the methanol extract obtained from fruiting bodies of *F. torulosa* led to the identification of eight compounds (**1**–**8**) (Figure 1). The concentrated methanol extract of *F. torulosa* was subjected to solvent–solvent partition between aqueous MeOH and *n*-hexane, followed by extraction with chloroform and ethyl acetate. The obtained organic solvent extracts were separated using a combination of flash column chromatography and reversed-phase HPLC, to give compounds **1**–**8**.

The ^1^H- and ^13^C-NMR spectra of compound **1** presented similar spectral features to those reported for gilvsin D [16] and obtained for natalic acid (**7**) [11]. Consecutive analysis of the COSY, HSQC, and HMBC spectra of **1** showed that it is a structural analogue of gilvsin D and natalic acid (**7**) and enabled the complete ^1^H- and ^13^C-NMR assignments listed in Table 1. On the basis of the spectral data similarities, all three compounds share the same degraded lanosterol skeleton. Structural differences were found in the side chains attached to C-17. HSQC and HMBC data suggested that in the case of compound **1,** a 22-hydroxy-24-en side chain was present. 1D and 2D ROESY data were in accordance with the suggested structure and proved that the relative configurations of C-4, C-5, C-10, C-13, C-14, C-17, and C-20 were identical in compound **1** and gilvsin D as well as in natalic acid (**7**). In addition to this, on the basis of the findings of González and coworkers [17] the ca. 3.6 Hz coupling constant observed between H-21 and H-22 suggested an *S* configuration of the C-22 chirality center. Putting these pieces of information together, compound **1** was characterized as 22*S*-hydroxy-8,24-dien-3-norlanosta-28-oic acid, known as fuscoporic acid (Figure 1).

Compounds **2** and **3** represent a mixture of inoscavin A and its *Z* isomer in a ca. 5 to 3 molar ratio. To the best of our knowledge the *cis* isomer (**3**) has not yet been reported in the literature. On the basis of the agreement of the obtained and published [18] NMR and HRMS data, the major component could be unambiguously assigned as inoscavin A. In accordance with the proposed structure, the minor component presented highly similar ^1^H and ^13^C-NMR features, excepting those belonging to the hispidine moiety of inoscavin A. Thus, in the ^1^H-NMR spectrum, instead of the two doublet resonances (δ_H_ 7.47 and 6.75 ppm) with 15.7 Hz coupling, two doublets at δ_H_ 6.87 and 6.11 ppm with a 12.7 Hz coupling constant were obtained for H-7 and H-6, respectively. This was in accordance with a C-6/C-7 double bond in *cis* configuration in the minor component. ^13^C-NMR, COSY, HSQC, and HMBC data confirmed the proposed structures and enabled the complete ^1^H and ^13^C-NMR assignments of both components (Figures S7–S10). Homonuclear ROESY data were also in accordance with these structural conclusions (Figure S11). 

**Figure 2:**
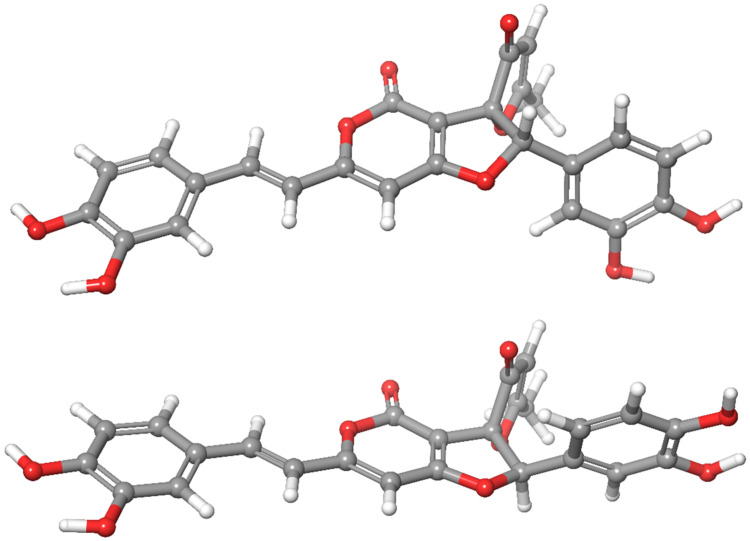
*SS* (**top**) and *RS* (**bottom**) epimers of inoscavin A (color scheme: grey: carbons, white: hydrogens, red: oxygens).

The relative stereochemistry of the C-4′ and C-5′ stereogenic centers could not be determined on this basis. Although inoscavin A has been known for quite some time, no literature data were found that could allow the stereochemical assignment of these centers on a comparative basis. Unfortunately, the amount of sample did not enable us to collect the specific experimental data (e.g., heteronuclear NOE) that might have allowed us to distinguish between the possible diastereoisomers. In the absence of adequate experimental data, a molecular modeling study was undertaken to determine the relative stereochemistry. Following the method described in the Experimental Section, NMR shielding constants and chemical shift values were calculated for the (arbitrary chosen) 4′*R*,5′*S* and 4′*S*,5′*S* epimers (Figure 2) by averaging the appropriate values obtained for four representative conformers using the Boltzmann populations derived from the solution phase energies (SI). As is shown in Table 2, the resulting unscaled chemical shifts (relative to TMS, using the default Jaguar procedure) are in very good agreement with the experimental data in both cases.

The mean absolute errors (MAE) calculated for ^13^C/^1^H chemical shifts were 1.2/0.17 and 1.3/0.26 ppm for the *SS* and *RS* epimers, respectively. The scaled shifts [19,20] were in even better agreement with the experimental values with (corrected) MAE values of ^13^C/^1^H 1.2/0.12 and 1.3/0.21 ppm for the SS* and RS* isomers, respectively. Although only small differences were obtained, these consistently pointed towards the presence of the SS* isomer. Recently Grimblat et al. [21] showed that the extended and combined use of the DP4 probability function introduced by Smith et al. [22] could successfully help to solve structural questions where other methods failed. Applying their DP4+ methodology [21] on the calculated isotropic shielding values (shieldings are listed in Table 2; DP4+ probabilities calculated by the template provided by the authors [21] are shown in Table 3), in our case, the *SS* isomer was predicted as the most probable (with a 100% overall possibility) structural candidate. On the basis of these results, compound **2** and **3** are described as the C-4′S*, C-5′S* isomers of inoscavin A and its *cis* analogue, respectively. Considering the obtained optical rotation value of [α${]}_{D}^{25}$ = 0 (*c*, 0.05, MeOH), the sample is a racemate. The isolation of **2** and **3** as a racemic mixture is in accordance with the finding of Kim et al. [18] who isolated inoscavin A for the first time, as well as with the nonstereoselective biogenetic pathway proposed for insocavin A by Mo et. al. [23]. It should be noted, however, that the small negative optical rotation value reported (without any discussion on the stereochemistry) for the close structural relative methylinoscavin A [24] “brings” some ambiguity to this question.

Compounds **4**–**8** reported here were structurally characterized on the basis of HRMS, and standard one- and two-dimensional NMR data in comparison to those reported in the literature. According to spectroscopical analysis, **4** and **5** represent an equimolar mixture of 3,4-dihydroxy-benzaldehide and osmundacetone. The remaining constituents are triterpenes, namely, senexdiolic acid (**6**) [17,25], natalic acid (**7**), and ergosta-7,22-diene-3-one (**8**) [26]. 

The identified fungal constituents were subjected to different pharmacological assays to determine their characteristic biological activities. In this way, the cytotoxic effect of **1**, **6**–**8** was tested on sensitive and resistant Colo 205 and Colo 320 colon adenocarcinoma cell lines, respectively, and on the normal MRC-5 embryonal fibroblast cell line with doxorubicin as a standard. While compounds **1**, **6,** and **7** did not show any significant effect in the applied concentration, compound **8** was effective against both Colo 205 (IC_50_ 11.65 ± 1.67 µM), Colo 320 (IC_50_ 8.43 ± 1.1 µM), and MRC-5 (IC_50_ 7.92 ± 1.42 µM) cell lines (Table 4). These results are comparable to those of doxorubicin (IC_50_ 2.46 ± 0.26 µM, 7.44 ± 0.2 µM and > 20 µM, respectively). 

After the promising results, a potentially synergistic relationship was investigated between **8** and doxorubicin (Figure S20). To this end, a checkerboard combination assay was utilized using the resistant Colo 320 cell line as a test subject. The results indicated that a 11.2:1 compound **8**: doxorubicin ratio was ideal for synergistic activity. At this rate, the combination index (CI) at the 50% growth inhibition dose (ED_50_) was 0.521±0.15, indicating a synergism between the examined compounds. 

The effect of compounds **1**, and **6**–**8** on the modulation of P-glycoprotein (P-gp) efflux was evaluated by flow cytometry, measuring the rhodamine-123 accumulation in MDR Colo 320 human colon adenocarcinoma cells (Figure S21). Tariquidar (0.2 µM), a well-known P-gp inhibitor, was used as positive control. The compounds were tested at 2 and 20 µM, and a P-gp modulating effect was obtained at a concentration of 2 µM with compound **8**, and at 20 µM in the case of compounds **1**, **6**, and **7**. The fluorescence activity ratio (FAR) values were used to assess the P-gp modulating potential. Usually, compounds can be considered to be active when presenting FAR values higher than 2. The results presented in Table 5 show that FAR values are in the range of 0.828–1.139; therefore, the tested compounds are not effective P-gp modulators on the drug-resistant strain Colo 320.

Fungal ergosterol derivatives, including glucosides, hydroxylated compounds, and peroxides, were demonstrated to have cytotoxic activity against human cancer cell lines other than those used in our assay. Furthermore, they exhibited strong inhibitory effects on cell proliferation in vitro, and apoptosis promoting and angiogenesis inhibitory activities in vivo [27,28,29]. Our results demonstrate for the first time that ergosta-7,22-diene-3-one (**8**) has potent anticancer activity against colon adenocarcinoma cells, which can be more effective in combination with the standard chemotherapeutic drug doxorubicin.

The antibacterial activity of the compounds was investigated using the broth dilution method. This method can provide precise numerical minimum inhibitory concentrations (MIC) data instead of the estimated antibacterial activity as is the case in the disk diffusion method. The antimicrobial effect of compounds **1**, and **6–8** were determined on *Escherichia coli* ATCC 25922, *Salmonella enterica* serovar Typhimurium 14028s, *Staphylococcus aureus* ATCC 25,923 and *S. aureus* 27,213 (methicillin and ofloxacin resistant clinical isolate) strains; however, none of the compounds produced a significant antibacterial effect (MIC > 100 µM).

The antioxidant capacity of compounds **2**+**3** and **4**+**5** was determined by DPPH and oxygen radical absorbance capacity (ORAC) assays. While both of the examined samples exhibited antioxidant effects in the two in vitro tests, **4**+**5** produced more promising results with 0.25 ± 0.01 µg/mL EC50 (DPPH) and 12.20 ± 0.92 mmol TE/g (ORAC), comparable to that of the reference compound, ascorbic acid (Table 6).

## 3. Materials and Methods

Optical rotations were measured with a Perkin-Elmer 341 polarimeter (PerkinElmer Life and Analytical Science, Shelton, CT, USA). The chemicals used in the experiments were supplied by Sigma-Aldrich, Hungary, and Molar Chemicals, Hungary. Flash chromatography was carried out on a CombiFlash^®^Rf+Lumen instrument with integrated UV, UV-Vis, and ELS detection using RediSep Rf Gold Normal Phase Silica Flash columns (4, 12 and 60 g) (Teledyne Isco, Lincoln, USA). Reversed-phase HPLC (RP-HPLC) separations were performed on a Wufeng LC-100 Plus HPLC instrument equipped with a UV-Vis detector (Shanghai Wufeng Scientific Instruments Co., Ltd., Shanghai, China) at 254 nm, using a Zorbax ODS column (250 × 4 mm, 5 µm; Agilent Technologies, Santa Clara, CA, USA). 

HRMS and MS analyses were performed on a Thermo Velos Pro Orbitrap Elite and Thermo LTQ XL (Thermo Fisher Scientific) system (Bremen, Germany). The ionization method was ESI operated in positive (or negative) ion mode. The (de)protonated molecular ion peaks were fragmented by CID at a normalized collision energy of 35%. For the CID experiment, helium was used as the collision gas. The samples were dissolved in methanol. Data acquisition and analysis were accomplished with Xcalibur software version 4.0 (Thermo Fisher Scientific, Bremen, Germany). NMR data were acquired on a Bruker Avance III HD 800 or 500 MHz NMR spectrometer (Bruker, Rheinstetten, Germany) both equipped with a TCI cold probe. MeOD-*d*_4_ or CDCl_3_ were used as solvents. Chemical shifts are reported in the delta scale relative to the residual solvent signals (3.31/7.27 and 49.15/77.0 ppm for ^1^H and ^13^C in MeOD/CDCl_3_, respectively). Standard one- and two-dimensional NMR spectra were recorded in all cases using the pulse sequences available in the TopSpin 3.5 sequence library. Data analysis and interpretation were performed with ACD/Labs 2017.1.3 NMR Workbook Suite. 

A molecular modeling study was performed within the Jaguar software package (Jaguar, version 10.4, Schrodinger, Inc., New York, NY, 2019) [30]. Firstly, a conformational search was performed at the molecular mechanics (MM) level using the default settings in MacroModel. After manual inspection of the resulted conformers, four representative conformers of each diastereomers were chosen for QM level geometry optimization using the Jaguar software package (Jaguar, version 10.4, Schrodinger, Inc., New York, NY, 2019) [30]. The gas phase geometry optimizations were performed at the B3LYP-D3/6-31+G** level while NMR shielding constants were calculated on the resulted geometries using a B3LYP functional and 6-311+G** basis set and PCM solvent model of methanol. The resulted isotropic shielding values of the conformers were Boltzmann averaged based on the calculated solution phase energies. Finally, DP4+ statistical analysis of these shieldings (with respect to the experimental chemical shifts) was carried out following the method and applying the template published by Grimblat, Zanardi, and Sarotti [21].

### 3.1. Mushroom Material

The mushroom samples were collected in March 2017 from Mt. Gerecse in the Central Transdanubia region, Hungary, and in April 2018 from the Botanical Garden of Buda. The former sample was found on Austrian oak, black locust, and small-leaved linden trees, the latter, however, was harvested from black locust unanimously. The fungal samples were combined for the chemical analysis. A voucher specimen was deposited in the mycological collection of Viktor Papp (PV1172).

### 3.2. Extraction and Isolation

The air-dried mushroom material (1.4 kg) was ground, then extracted with MeOH (20 L) at room temperature. After concentration, the MeOH extract (44.26 g) was dissolved in 50% aqueous MeOH and subjected to solvent–solvent partition with *n*-hexane (5 × 300 mL), chloroform (6 × 300 mL), and ethyl acetate (6 × 300 mL). The *n*-hexane fraction (10.34 g) was subjected to flash chromatography on a silica gel column using a gradient system of *n*-hexane and acetone (0%–40%; *t* = 55 min). Fractions with similar compositions were combined according to TLC monitoring (A1–A10). The combined fractions A2 and A3 (0.80 g) were purified by flash chromatography using a mixture of *n*-hexane and acetone (0–25%; *t* = 50 min), with increasing polarity, to obtain compound **7** (2.9 mg). Fractions A4 and A5 (4.99 g) were further separated by multiple flash chromatography steps, applying *n*-hexane–acetone and H_2_O-MeOH solvent systems on normal and reversed phase stationary phases, respectively, then a final purification was performed by RP-HPLC using a H_2_O-MeOH gradient system to give compounds **1** (14.4 mg) and **6** (9.6 mg). 

The chloroform soluble phase (13.47 g) was subjected to flash chromatography in multiple steps on silica gel column using gradient system of *n*-hexane–acetone. Fractions with similar compositions were combined according to TLC monitoring (B1-B13). Fractions B6-B8 (2.16 g) were further separated by combination of flash chromatography (*n*-hexane–acetone 5% to 25%, *t* = 50 min) to obtain an equimolar mixture of **3** and **4** (62.3 mg) and **5** (4.7 mg). 

Finally, the ethyl acetate phase (10.60 g) was further separated in subsequent flash chromatography steps, then fractions with related compositions were combined according to TLC monitoring (B1-B9). The fractionation of C2-4 (0.62 g) by normal phase flash chromatography using a chloroform–MeOH system (0–40%, *t* = 50 min) led to the isolation of **2** (1.4 mg).

Fuscoporic acid (**1**): a white, amorphous solid; [α${]}_{D}^{25}$ + 28 (MeOH, *c* 0.1), ^1^H and ^13^C-NMR data are shown in in Table 1; HRMS: [M-H]^−^ 441.33698 (*δ* = −1.0 ppm; C_29_H_45_O_3_). HR-ESI-MS-MS (CID = 45%; rel. int. %): 411(36); 371(100).

### 3.3. Cell Culture

The human colon adenocarcinoma cell lines, the Colo 205 (ATCC-CCL-222) doxorubicin-sensitive parent and Colo 320/MDR-LRP (ATCC-CCL-220.1) resistant to anticancer agents expressing ABCB1, were purchased from LGC Promochem (Teddington, UK). The cells were cultured in RPMI-1640 medium supplemented with 10% heat-inactivated fetal bovine serum (FBS), 2 mM L-glutamine, 1 mM Na-pyruvate, 100 mM Hepes, nystatin, and a penicillin–streptomycin mixture in concentrations of 100 U/L and 10 mg/L, respectively. The MRC-5 (ATCC CCL-171) human embryonic lung fibroblast cell line (LGC Promochem) was cultured in EMEM medium, supplemented with 1% nonessential amino acid (NEAA) mixture, 10% heat-inactivated FBS, 2 mM L-glutamine, 1 mM Na-pyruvate, nystatin, and a penicillin–streptomycin mixture in concentrations of 100 U/L and 10 mg/L, respectively. The cell lines were incubated in a humidified atmosphere (5% CO_2_, 95% air) at 37 °C.

### 3.4. Assay for Cytotoxic Effect

The effects of increasing concentrations of the compounds on cell growth were tested in 96-well flat-bottomed microtiter plates [31]. The two-fold serial dilutions of the tested compounds were made starting with 100 μM. Then, 10^4^ of human colonic adenocarcinoma cells in 100 μL of the medium (RPMI-1640) were added to each well, except for the medium control wells. The adherent human embryonic lung fibroblast cell line (10^4^/well) was seeded in EMEM medium in 96-well flat-bottomed microtiter plates for 4 h before the assay. The serial dilutions of the compounds were made in a separate plate starting with 100 μM, and then transferred to the plates containing the adherent corresponding cell line. Culture plates were incubated at 37 °C for 24 h; at the end of the incubation period, 20 μL of MTT (thiazolyl blue tetrazolium bromide) solution (from a 5 mg/mL stock solution) were added to each well. After incubation at 37 °C for 4 h, 100 μL of sodium dodecyl sulfate (SDS) solution (10% SDS in 0.01 M HCl) were added to each well and the plates were further incubated at 37 °C overnight. Cell growth was determined by measuring the optical density (OD) at 540 nm (ref. 630 nm) with a Multiscan EX ELISA reader (Thermo Labsystems, Cheshire, WA, USA). Inhibition of cell growth was expressed as IC_50_ values, defined as the inhibitory dose that reduces the growth of the cells exposed to the tested compounds by 50%. IC_50_ values and the SD of triplicate experiments were calculated using GraphPad Prism software version 5.00 for Windows with nonlinear regression curve fit (GraphPad Software, San Diego, CA, USA; www.graphpad.com). The statistical analysis of data was performed using GraphPad Prism software version 5.00, applying the two-tailed *t*-test.

### 3.5. Checkerboard Combination Assay

A checkerboard microplate method [32] was applied to study the effect of drug interactions between the compound **8** and the chemotherapeutic drug doxorubicin. The assay was carried out on Colo 320 colon adenocarcinoma cell line. The final concentration of the compounds and doxorubicin used in the combination experiment was chosen in accordance with their cytotoxicity towards this cell line. The dilutions of doxorubicin were made in a horizontal direction for the 100 μL volume, and the dilutions of the compounds were made vertically in the microtiter plate for the 50 μL volume. Then, 6 × 10^3^ of Colo 320 cells in 50 μL of the medium were added to each well, except for the medium control wells. The plates were incubated for 72 h at 37 °C in 5% CO_2_ atmosphere. The cell growth rate was determined after MTT staining. At the end of the incubation period, 20 μL of MTT solution (from a stock solution of 5 mg/mL) were added to each well. After incubation at 37 °C for 4 h, 100 μL of SDS solution (10% in 0.01 M HCI) were added to each well and the plates were further incubated at 37 °C overnight. OD was measured at 540 nm (ref. 630 nm) with a Multiscan EX ELISA reader. Combination index (CI) values at 50% of the growth inhibition dose (ED_50_) were determined using CompuSyn software (ComboSyn, Inc., Paramus, NJ, USA) to plot four to five data points at each ratio. CI values were calculated by means of the median-effect equation, according to the Chou–Talalay method, where CI < 1, CI = 1, and CI > 1 represent synergism, additive effect (or no interaction), and antagonism, respectively [33,34].

### 3.6. Rhodamine 123 Accumulation Assay

The cell numbers of the human colon adenocarcinoma cell lines were adjusted to 2 × 10^6^ cells/mL, re-suspended in serum-free RPMI 1640 medium, and distributed in 0.5 mL aliquots into Eppendorf centrifuge tubes. The tested compounds were added at concentrations of 2 or 20 μM, and the samples were incubated for 10 min at room temperature. Tariquidar was applied as positive control at 0.2 μM. DMSO at 2% *v*/*v* was used as solvent control. Next, 10 µL (5.2 µM final concentration) of the fluorochrome and ABCB1 substrate rhodamine 123 (Sigma) were added to the samples and the cells were incubated for a further 20 min at 37 °C, washed twice, and re-suspended in 1 mL PBS for analysis. The fluorescence of the cell population was measured with a PartecCyFlow^®^ flow cytometer (Partec, Münster, Germany). The fluorescence activity ratio was calculated as the quotient between the FL-1 of the treated/untreated resistant Colo 320 cell line over the treated/untreated sensitive Colo 205 cell line according to the following equation [31]:

(1) $$\mathrm{FAR}\;=\;\frac{{\mathrm{Colo}320}_{\mathrm{treated}}{/\mathrm{Colo}320}_{\mathrm{control}}}{{\mathrm{Colo}205}_{\mathrm{treated}}{/\mathrm{Colo}205}_{\mathrm{control}}}$$

### 3.7. Bacterial Strains

*Escherichia coli* ATCC (American Type Culture Collection) 25922, *Salmonella enterica* serovar Typhimurium 14028s, *Staphylococcus aureus* ATCC 25,923, and the methicillin and ofloxacin resistant *S. aureus* 272,123 clinical isolates were used in the study.

### 3.8. Determination of Minimum Inhibitory Concentrations by Microdilution Method

The minimum inhibitory concentrations (MICs) of all tested compounds were determined according to the Clinical and Laboratory Standards Institute (CLSI) guidelines in three independent assays. The compounds were diluted in 100 μL of Mueller–Hinton medium in 96-well flat-bottomed microtiter plates. The starting concentration was 100 μM, and two-fold serial dilutions were prepared in the microplates before the addition of the bacterial culture. Then, a 10^−4^ dilution of an overnight bacterial culture in 100 μL of the medium was added to each well, with the exception of the medium control wells. The plates were further incubated at 37 °C for 18 h; at the end of the incubation period, MIC values of the tested compounds were determined by naked eye [35].

### 3.9. DPPH Assay

A method based on the description of Miser-Salihoglu E. et al. was applied [36]. The examination was performed on a FLUOstar Optima BMG Labtech plate-reader with 96-well microplates. The samples were measured in a DMSO environment with the volume of 150 µL per sample resulting 1 mg/mL concentration. Every well contained 50 µL (100 µM) of this base solution for the absorbance measurement (30 min; 550 nm). When samples showed no or minor activity, the concentration was doubled for a follow-up measurement. For the most active samples, half the maximal effective concentration (EC_50_) was determined using a dilution series beginning with a 100 µM solution which was halved at every consecutive step. For data evaluation GraphPad Prism 6.0 software was utilized. The DPPH (2,2′-diphenyl-1-picrylhydrazyl) reagent necessary for the process was supplied by from Sigma-Aldrich Hungary [4].

### 3.10. ORAC Assay

A method based on the description of Mielnik et al. was applied [37]. In summary, 20 µL of the samples were used in 0.01 mg/mL concentration with a 96-well black microplate. In each well, 60 µL of AAPH (12 mM final concentration) and 120 µL of fluorescein solution (70 nM final concentration) were added. The fluorescence alteration of each sample was measured for 3 h with 1.5 min cycle intervals by a FLUOstar Optima BMG Labtech plate-reader. Trolox ((±)-6-hydroxy-2,5,7,8-tetramethyl-chromane-2-carboxylic acid) was used as a standard. Both Trolox and AAPH was supplied by Sigma-Aldrich, Hungary, while Fluorescein was provided by Fluka analytical, Japan. For data analysis, the GraphPad Prism 6.0 software was used. The results were expressed as mmol Trolox equivalent per g of dry material (mmloTE/g) [4].

## 4. Conclusions

The present report highlights the most important results acquired upon a detailed chemical analysis of *Fuscoporia torulosa.* These represent a valuable addition to the biological activity studies of this species. The combination of chromatographic methods led to the identification of six compounds including the novel fuscoporic acid (**1**) and Z- inoscavin A. Biological activity (cytotoxicity, synergistic, MDR reversal, antioxidant, and antibacterial) assays were performed to explore the pharmacological potential of the chemical constituents of this fungus. The results obtained revealed that ergosta-7,22-diene-3-one (**8**) not only exhibits a considerable cytotoxic effect on human colon adenocarcinoma cell lines, but also exhibits synergism with the reference compound doxorubicin**.** In summary, this study provides notable evidence for the increased interest in the polypore *F. torulosa* as a source of fungal metabolites with antioxidant and cytotoxic properties.

## Figures and Tables

**Table 1:** ^1^H- and ^13^C-NMR assignments of **1.**

Position	δ^13^C ppm	δ^1^H ppm	Multiplicity (*J* in Hz)
1 α	36.0	1.49	m
1 β		1.60	m
2 α	36.9	2.48	dd (13.7, 8.3)
2 β		1.67	m
4	48.2		
5	52.9	2.06	m
6 α		1.63	m
6 β	18.6 25.9	1.72	m
7		2.12	m
8	134.1		
9	135.0		
10	45.6		
11 α	22.5	2.02	m
11 β	30.4	2.13	m
12 α		1.76	m
12 β		1.69	m
13	45.3		
14	48.9		
15 α	30.6	1.20	m
15 β	27.3	1.60	m
16 α		1.82	m
16 β	47.1	1.44	m
17		1.57	m
18	15.5	0.76	s
19	19.3	0.98	s
20	41.6	1.80	m
21	12.7	0.96	d (6.6)
22	73.4	3.68	m
23	29.1	2.05	m
24	121.3	5.19	m
25	135.2		
26	26.0	1.75	s
27	18.0	1.66	s
28	185.3		
29	21.3	1.24	s
30	24.4	0.89	s

**Table 2:** Experimental and calculated chemical shifts and isotropic shielding values of *S,S* and *R,S* isomers.

No.	Atom No. in Figure 1	δ_exp_ Experimental Shift (ppm)	*SS* Boltzmann Averaged Shielding	*RS* Boltzmann Averaged Shielding	δcalc *SS* Uncaled Shift (ppm) *	δs *SS* Scaled Shift (ppm)	δcalc *RS* Unscaled Shift (ppm) *	δs *RS* Scaled Shift (ppm)
C1	Me	16.8	165.1	164.5	16.2	16.3	16.8	16.3
C2	1′	193.1	−18.1	−18.5	195.1	195.1	195.5	195.8
C3	2′	105.3	74.1	74.8	105.1	105.2	104.4	104.3
C4	3′	203.3	−25.1	−24.1	202.0	202.0	201.0	201.3
C6	4′	94.4	82.5	80.5	96.9	97.0	98.8	98.7
C8	5′	96.0	83.0	85.5	96.4	96.5	93.9	93.7
C10	3	177.0	0.6	0.8	176.9	176.9	176.6	176.9
C11	2	99.6	80.7	79.5	98.6	98.7	99.8	99.7
C12	1	160.8	19.3	18.9	158.6	158.7	159.0	159.1
C15	5	167.2	9.0	9.2	168.6	168.7	168.4	168.6
C16	4	95.7	84.1	83.8	95.3	95.4	95.6	95.4
C17	6	116.8	62.3	62.5	116.6	116.6	116.4	116.4
C18	7	140.7	36.6	36.8	141.7	141.8	141.5	141.6
C19	8	128.6	48.5	48.8	130.0	130.1	129.8	129.8
C20	9	115.2	64.5	64.3	114.4	114.5	114.7	114.6
C21	10	147.1	31.2	31.2	146.9	147.0	147.0	147.0
C22	11	149.6	30.3	30.4	147.9	147.9	147.7	147.8
C23	12	116.1	63.7	63.7	115.3	115.3	115.2	115.2
C24	13	122.8	54.3	54.5	124.4	124.5	124.2	124.2
C27	6′	123.3	52.1	51.4	126.6	126.7	127.2	127.2
C28	7′	115.6	64.0	62.9	114.9	115.0	116.0	115.9
C29	8′	146.4	31.6	32.0	146.6	146.7	146.2	146.3
C30	9′	148.0	31.9	32.1	146.3	146.4	146.1	146.2
C31	10′	116.8	65.2	65.3	113.8	113.9	113.7	113.6
C32	11′	120.4	59.2	57.8	119.6	119.7	121.0	121.0
H35	5′	5.68	26.27	25.92	5.56	5.51	5.90	5.75
H36	6	6.75	24.88	24.98	6.90	6.80	6.80	6.67
H37	7	7.47	23.99	23.93	7.75	7.61	7.80	7.71
	Me	2.00	29.95	29.54	2.03	2.13	2.43	2.20
H41	2′	5.60	26.33	26.65	5.51	5.46	5.20	5.03
H42	4	6.53	25.53	25.64	6.27	6.19	6.17	6.03
H43	9	7.10	24.30	24.31	7.45	7.32	7.44	7.33
H44	12	6.78	24.87	24.86	6.90	6.80	6.92	6.80
H45	13	7.02	24.54	24.54	7.22	7.10	7.22	7.11
H48	7′	6.73	24.83	24.67	6.94	6.84	7.10	6.99
H49	10′	6.82	24.94	24.94	6.84	6.74	6.84	6.72
H50	11′	6.61	25.08	24.88	6.70	6.61	6.89	6.77

* δ_calc_ unscaled shift are calculated by Jaguar relative to TMS (based on a semiempirical linear regression against experimental data); δ_s_ scaled shifts are calculated according to δ_s_ = (δ_calc_ − b)/m, where b and m are the intercept and slope of a plot of δ_calc_ against δ_exp._

**Table 3:** DP4+ probabilities obtained for *SS* and *RS* isomers of **2** using the template from [21]; s and u refer to scaled and unscaled shifts.

	*SS*	*RS*
sDP4+ (H data)	99.87%	0.13%
sDP4+ (C data)	96.73%	4.27%
sDP4+ (all data)	100%	0%
uDP4+ (H data)	100%	0%
uDP4+ (C data)	79.89%	20.11%
uDP4+ (all data)	100%	0%
DP4+ (H data)	100%	0%
DP4+ (C data)	99.16%	0.84%
DP4+ (all data)	100%	0%

**Table 4:** Cytotoxic activity of compounds **1**, **6**–**8,** and doxorubicin on the human colon adenocarcinoma cell lines (sensitive Colo 205 and resistant Colo 320 cells) and on the normal MRC-5 embryonal fibroblast cell line.

Samples	IC_50_ (µM)		
	Colo 205	Colo 320	MRC-5
**1**	>100	>100	>100
**6**	>100	>100	>100
**7**	>100	>100	>100
**8**	11.65 ± 1.67 ***	8.43 ± 1.1	7.92 ± 1.42 **
Doxorubicin	2.46 ± 0.26	7.44 ± 0.2	> 20

** *p* < 0.01, *** *p* < 0.001.

**Table 5:** Effect of compounds **1**, **6**–**8** on the P-glycoprotein (P-gp)-mediated rhodamine-123 efflux on MDR human colon adenocarcinoma (Colo 320).

Samples	conc. (μM)	FSC	SSC	FL-1	FAR
Tariquidar	0.2	1945	837	64.100	5.533
**1**	20	2005	851	13.200	1.139
**6**	20	2074	861	11.900	1.027
**7**	20	2095	891	12.200	1.053
**8**	2	2099	857	10.100	0.872
DMSO	2.00%	2073	848	9.590	0.828
Colo 320	-	2052	841	8.870	-

Parameters evaluated from flow cytometric experiments were forward scatter count (FSC, provides information about cell size); side scatter count (SSC, proportional to cell granularity or internal complexity); FL-1 (mean fluorescence of the cells), and fluorescence activity ratio (FAR), which was calculated by the equation given in the Section 3. The histograms were evaluated regarding the mean fluorescence intensity, the standard deviation of 20,000 individual cells belonging to the total, and the gated populations.

**Table 6:** Antioxidant activity of compounds **2**–**5**.

Compounds	DPPH EC_50_ (µg/mL)	ORAC Activity (mmol TE/g)
**2+3**	0.72 ± 0.05	2.70 ± 0.03
**4+5**	0.25 ± 0.01	12.20 ± 0.92
Ascorbic acid	0.89 ± 0.02	6.94 ± 0.58

## Data Availability

The data presented in this study are available in this article.

## Supplementary Materials

The following are available online, Figure S1. 800 MHz ^1^H NMR spectrum of compound **1**, Figure S2. 200 MHz ^13^C NMR spectrum of compound **1**. Figure S3. 800 MHz HSQC spectrum of compound **1**, Figure S4. 800 MHz HMBC spectrum of compound **1**, Figure S5. 800 MHz ROESY spectrum of compound **1**, Figure S6. Literature structure of compound **2**, Figure S7. 800 MHz ^1^H NMR spectrum of mixture of compounds **2** and **3**, Figure S8. 800 MHz ^13^C NMR spectrum of mixture of compounds **2** and **3**, Figure S9. 800 MHz HMBC spectrum of mixture of compounds **2** and **3**, Figure S10. 800 MHz HSQC spectrum of mixture of compounds **2** and **3**, Figure S11. 800 MHz ROESY spectrum of mixture of compounds **2** and **3**, Figure S12. 200 MHz ^13^C NMR spectrum of mixture of compounds **4** and **5**, Figure S13. 800 MHz ^1^H NMR spectrum mixture of compounds **4** and **5**, Figure S14. 800 MHz ^1^H NMR spectrum of compound **6**, Figure S15. 200 MHz ^13^C NMR spectrum of compound **6**, Figure S16. 500 MHz ^1^H NMR spectrum of compound **7**, Figure S17. 125 MHz ^13^C NMR spectrum of compound **7**, Figure S18. 500 MHz ^1^H NMR spectrum of compound **8**, Figure S19. 125 MHz ^13^C NMR spectrum of compound **8**, Figure S20. Cytotoxicity dose-effect curve of compound **8**, doxorubicin and their combination on Colo 320 cell line, report of the combination assay of compound **8**, Figure S21. Histograms of modulation of P-gp efflux pump assay. Table S1. Isotropic shieldings and unscaled chemical shifts of the conformers of *SS* isomer of **2** and **3**, Table S2. Isotropic shieldings and unscaled chemical shifts of the conformers of *SR* isomer of **2** and **3**, Table S3. xyz coordinates of conformers of **2** and **3**.

## Sample Availability

Samples of the compounds **1**, **3**–**7** are available from the authors.
