# Supplementary material for: Triterpenes and Phenolic Compounds from the Fungus Fuscoporia torulosa: Isolation, Structure Determination and Biological Activity
Source: Molecules. 2021 Mar 16;26(6):1657. doi: 10.3390/molecules26061657 (PMC8002351; doi:10.3390/molecules26061657)
Supplement: Supplementary file 1 [file molecules-26-01657-s001.zip › molecules-1120261-supplementary.pdf]

Triterpenes and phenolic compounds from the fungus *Fuscoporia torulosa*: isolation, structure determination and biological activity

Zoltán Béni [1], Miklós Dékány [1], András Sárközy [2], Annamária Kincses [3], Gabriella Spengler [3], Viktor Papp [4], Judit Hohmann [2,5*], Attila Ványolós [2,6*]

[1] *Spectroscopic Research, Gedeon Richter Plc., Gyömrői út 19-21, H-1103 Budapest, Hungary; z.beni@richter.hu, M.Dekany@richter.hu*

[2] *Department of Pharmacognosy, University of Szeged, Eötvös u. 6, H-6720 Szeged, Hungary; sarkozy@pharmacognosy.hu*

[3] *Department of Medical Microbiology and Immunobiology, University of Szeged, Dóm square 10, H-6720, Szeged, Hungary; spengler.gabriella@med.u-szeged.hu, kincses.annamaria@med.u-szeged.hu*

[4] *Hungarian University of Agriculture and Life Sciences, Department of Botany Department of Botany, Szent István University, H-1118 Budapest, Villányi út 29-43, Hungary; papp.viktor@uni-mate.hu*

[5] *Interdisciplinary Centre for Natural Products, University of Szeged, Eötvös u. 6, H-6720 Szeged, Hungary; hohmann.judit@szte.hu*

[6] *Department of Pharmacognosy, Semmelweis University, Üllői u. 26, H-1085 Budapest, Hungary; vanyolosa@pharmacognosy.hu*

*****Correspondence: hohmann.judit@szte.hu; hohmann.judit@szte.hu; Tel.: +36-62-545 558

TABLE OF CONTENT

Spectra and spectral data on compound 1 ..... **3**

Figure S1. 800 MHz ^{1}H NMR spectrum of compound **1** 3

Figure S2. 200 MHz ^{13}C NMR spectrum of compound **1** 3

Figure S3. 800 MHz HSQC spectrum of compound **1** 4

Figure S4. 800 MHz HMBC spectrum of compound **1** 5

Figure S5. 800 MHz ROESY spectrum of compound **1** 6

Spectra and spectral data on compounds 2 and 3 ..... **7**

Table S1. Isotropic shieldings and unscaled chemical shifts of the conforemers of *SS* isomer 7

Table S2. Isotropic shieldings and unscaled chemical shifts of the conforemers of *SR* isomer 8

Table S3. xyz coordinates of conformers 10

Figure S6. Literature structure of compound **2**[1] 18

Figure S7. 800 MHz ^{1}H NMR spectrum of mixture of compounds **2** and **3** 19

Figure S8. 800 MHz ^{13}C NMR spectrum of mixture of compounds **2** and **3** 20

Figure S9. 800 MHz HMBC spectrum of mixture of compounds **2** and **3** 21

Figure S10. 800 MHz HSQC spectrum of mixture of compounds **2** and **3** 22

Figure S11. 800 MHz ROESY spectrum of mixture of compounds **2** and **3** 23

Spectra and spectral data on compounds 4 and 5 ..... **24**

Figure S12. 200 MHz ^{13}C NMR spectrum of mixture of compounds **4** and **5** 25

Figure S13. 800 MHz ^{1}H NMR spectrum mixture of compounds **4** and **5** 25

Spectra and spectral data on compound 6 ..... **26**

Figure S14. 800 MHz ^{1}H NMR spectrum of compound **6** 26

Figure S15. 200 MHz ^{13}C NMR spectrum of compound **6** 27

Spectra and spectral data on compound 7 ..... **28**

Figure S16. 500 MHz ^{1}H NMR spectrum of compound **7** 28íí

Figure S17. 125 MHz ^{13}C NMR spectrum of compound **7** 29

Spectra and spectral data on compound 8 30

Figure S18. 500 MHz ^{1}H NMR spectrum of compound **8** 30

Figure S19. 125 MHz ^{13}C NMR spectrum of compound **8** 31

Data of cytotoxicity and P-gp inhibitory assays **32**

Figure S20. Cytotoxicity dose-effect curve of compound **8**, doxorubicin and their combination on Colo 320 cell line 32

Report of the combination assay of compound **8** 33

Figure S21. Histograms of modulation of P-gp efflux pump assay 37

References **39**

Spectra and spectral data on compound 1

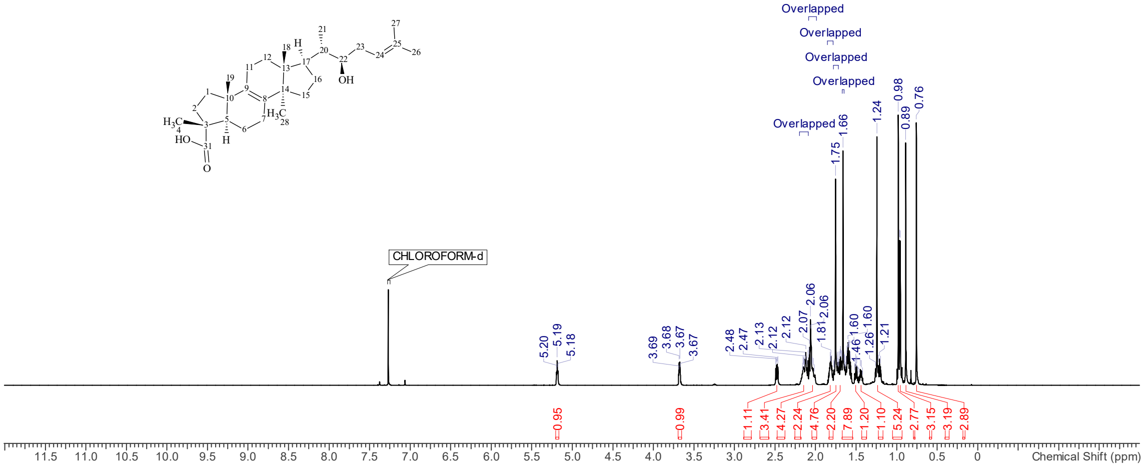

Figure S1. 800 MHz ^{1}H NMR spectrum of compound **1**

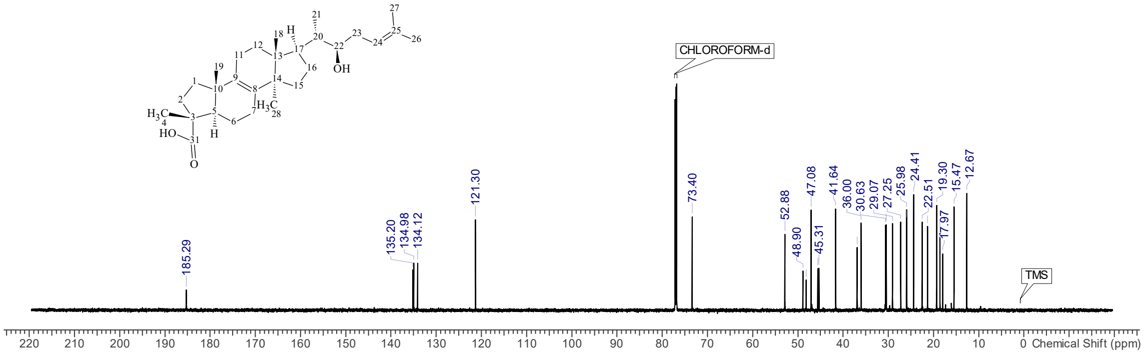

Figure S2. 200 MHz ^{13}C NMR spectrum of compound **1**

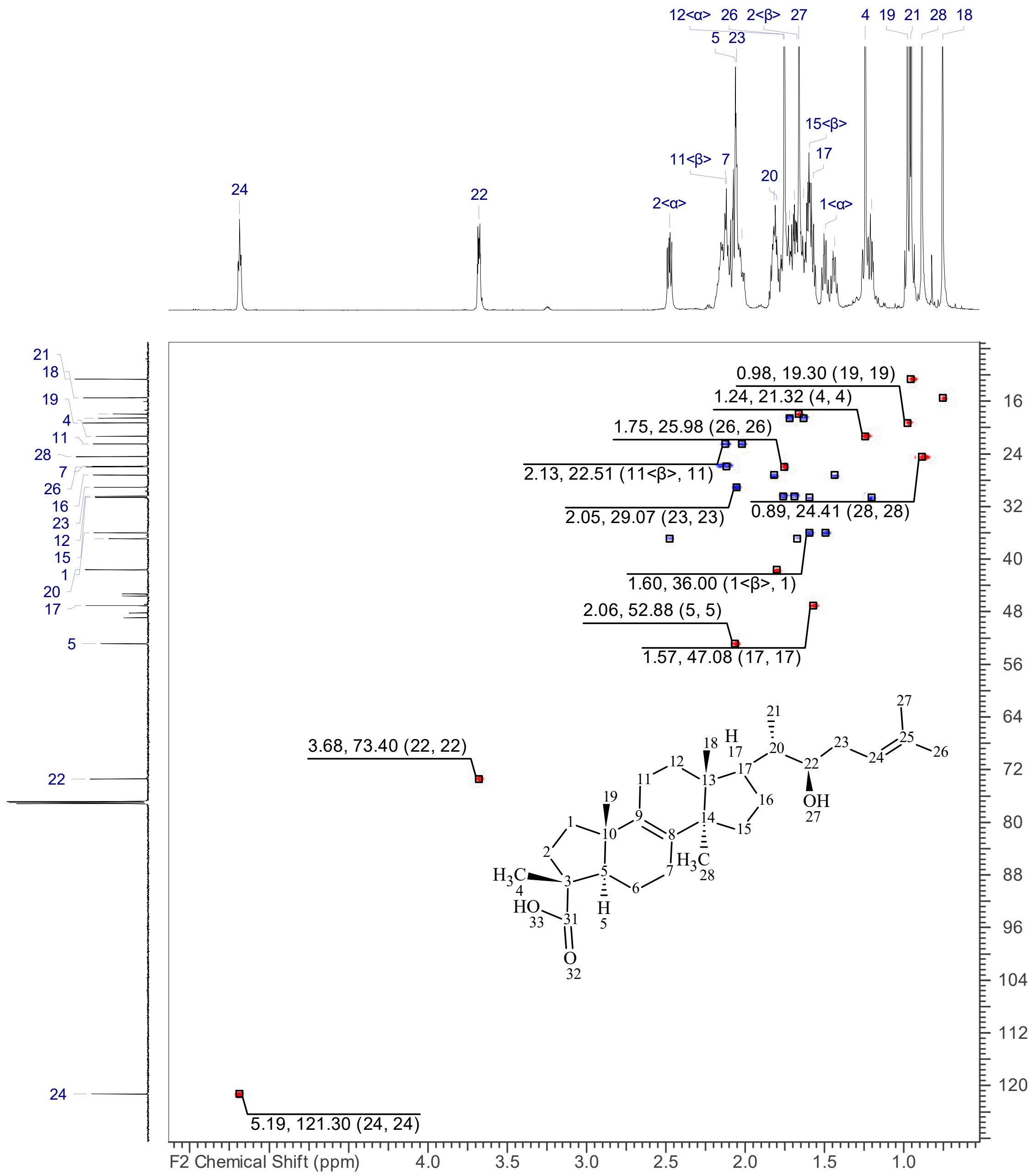

Figure S3. 800 MHz HSQC spectrum of compound **1**

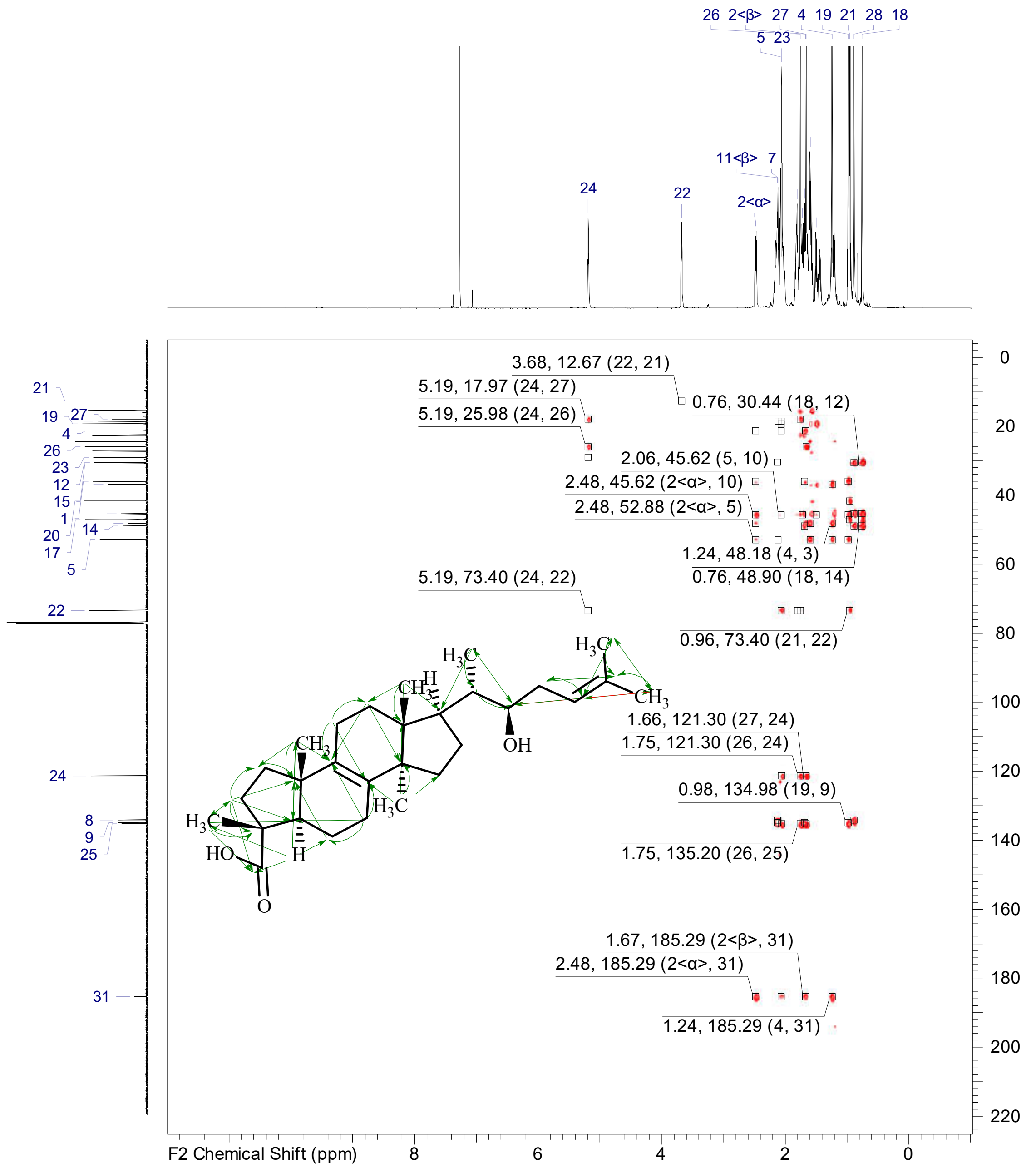

Figure S4. 800 MHz HMBC spectrum of compound **1**

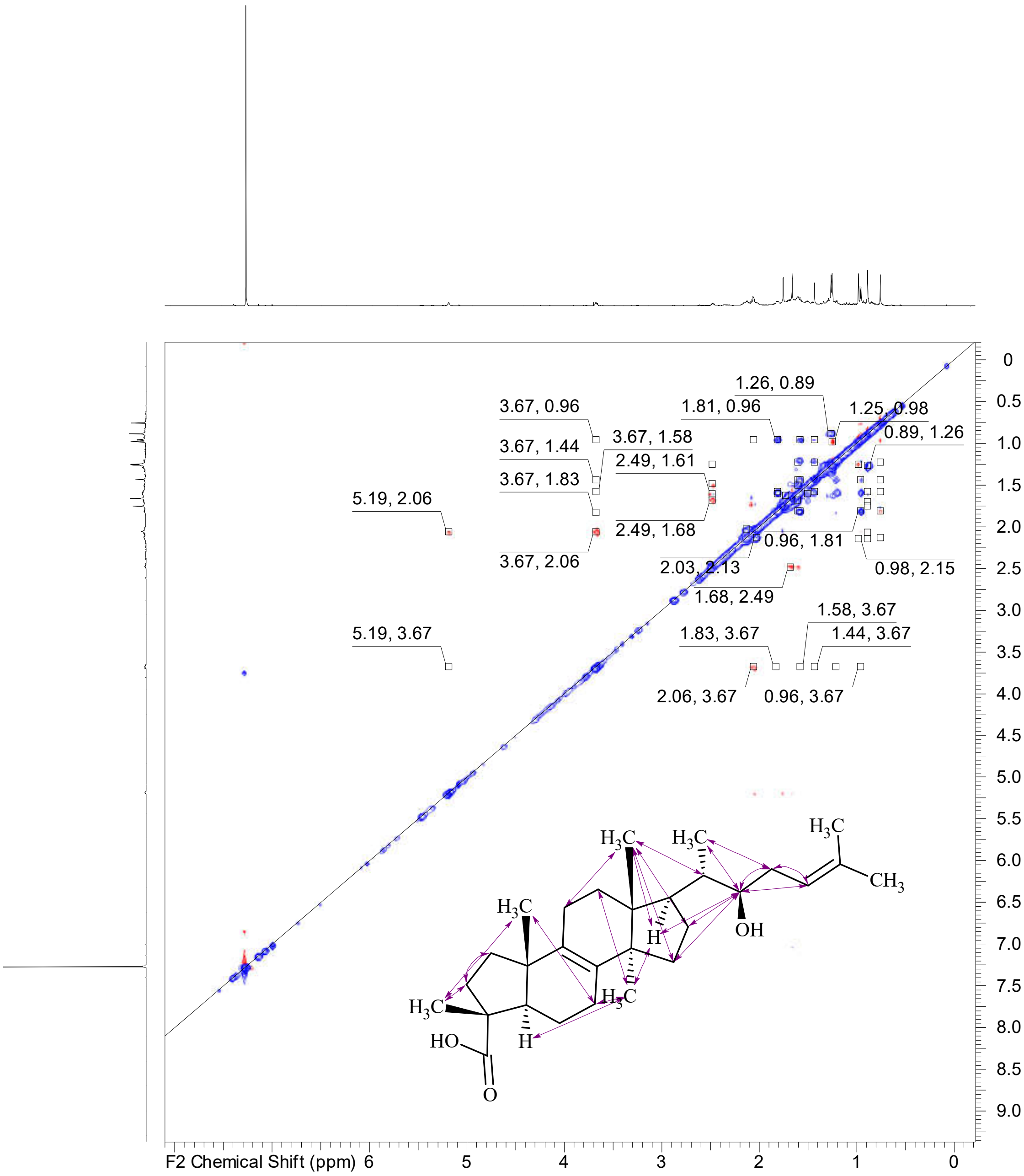

Figure S5. 800 MHz ROESY spectrum of compound **1**

Spectra and spectral data on compounds 2 and 3

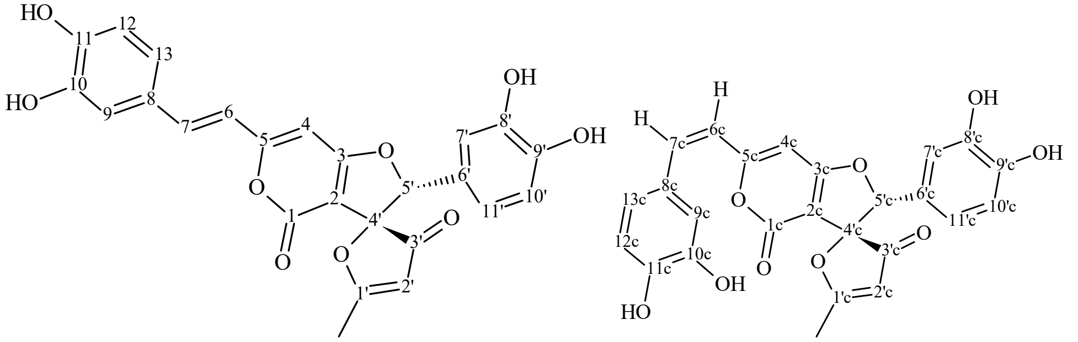

Structure and numbering of Inoscavin A, only one enantiomer is shown for clarity

Table S1. Isotropic shieldings and unscaled chemical shifts of the conforemers of *SS* isomer

	shielding SS_1_MeOH	δχalc SS_1_MeOH	shielding SS_2_MEOH	δcalc SS_2_MEOH	shielding SS_3_MEOH	δcalc SS_3_MEOH	shielding SS_4_MEOH	δcalc SS_4_MEOH
Boltzmann population	42.486		24.422		22.867		10.227	
C1	165.07	16.22	165.14	16.15	165.13	16.16	165.16	16.13
C2	-18.07	195.08	-17.92	194.94	-18.29	195.3	-18.13	195.14
C3	74.2	104.96	74.16	105	73.97	105.19	73.72	105.43
C4	-25.07	201.91	-24.88	201.73	-25.43	202.27	-25.1	201.94
C6	82.64	96.72	82.56	96.8	82.17	97.18	82.17	97.18
C8	82.81	96.56	82.73	96.63	83.46	95.92	83.26	96.12
C10	0.48	176.96	0.59	176.86	0.70	176.75	0.61	176.84
C11	80.67	98.65	80.73	98.59	80.77	98.54	80.8	98.52
C12	19.26	158.62	19.33	158.55	19.3	158.58	19.33	158.56
C15	8.91	168.73	9.18	168.47	8.96	168.68	9.06	168.58
C16	84.17	95.23	83.99	95.4	84.24	95.15	84.15	95.25
C17	62.56	116.33	61.88	117.00	62.57	116.32	61.95	116.93
C18	36.43	141.85	36.76	141.53	36.78	141.51	36.57	141.72
C19	48.49	130.08	48.63	129.94	48.48	130.08	48.65	129.92
C20	67.86	111.15	58.33	120.46	67.72	111.29	58.35	120.44
C21	30.98	147.17	31.69	146.48	30.94	147.21	31.74	146.44
C22	30.09	148.05	30.66	147.48	30.12	148.02	30.6	147.55
C23	63.99	114.93	63.1	115.81	63.89	115.04	63.13	115.78
C24	50.79	127.83	60.77	118.08	51.12	127.5	60.63	118.21
C27	52.5	126.16	52.6	126.06	51.08	127.55	51.29	127.34
C28	62.98	115.93	62.77	116.13	66.36	112.62	66.36	112.62
C29	31.45	146.72	31.45	146.71	31.78	146.39	31.78	146.39
C30	31.57	146.60	31.54	146.62	32.51	145.68	32.56	145.63
C31	65.43	113.53	65.49	113.47	64.57	114.37	64.59	114.35
C32	58.62	120.18	58.43	120.37	60.46	118.38	60.48	118.36
H35	26.3	5.53	26.3	5.53	26.2	5.63	26.22	5.61
H36	24.89	6.89	24.83	6.94	24.91	6.87	24.86	6.92

H37	23.93	7.8	24.06	7.68	23.99	7.75	24.06	7.69
Me	29.94	2.05	29.93	2.05	29.98	2	29.99	1.99
H41	26.37	5.47	26.37	5.47	26.24	5.59	26.22	5.61
H42	25.53	6.27	25.55	6.25	25.51	6.29	25.53	6.27
H43	24.13	7.61	24.66	7.11	24.08	7.66	24.66	7.11
H44	24.89	6.88	24.82	6.96	24.91	6.87	24.83	6.94
H45	24.75	7.02	24.12	7.62	24.78	6.99	24.17	7.58
H48	24.62	7.14	24.62	7.14	25.24	6.55	25.26	6.53
H49	25	6.78	25	6.78	24.82	6.96	24.82	6.95
H50	25.25	6.54	25.25	6.54	24.73	7.04	24.74	7.03

Table S2. Isotropic shieldings and unscaled chemical shifts of the conformers of *SR* isomer

	shielding SR_1_MEOH	δcalc SR_1_MEOH	shielding SR_2_MEOH	δcalc SR_2_MEOH	shielding SR_3_MEOH	δcalc SR_3_MEOH	shielding SR_4_MEOH	δcalc SR_4_MEOH
Boltzmann population	34.994		21.178		27.966		15.862	
C1	164.51	16.76	164.53	16.74	164.47	16.8	164.56	16.71
C2	-18.45	195.45	-18.32	195.32	-18.58	195.58	-18.63	195.63
C3	74.79	104.39	74.82	104.36	74.75	104.43	74.75	104.42
C4	-24.21	201.08	-24.19	201.06	-24.02	200.89	-24.08	200.95
C6	80.42	98.89	80.26	99.05	80.63	98.68	80.55	98.76
C8	85.47	93.95	85.5	93.93	85.56	93.87	85.62	93.81
C10	0.79	176.66	0.91	176.55	0.82	176.63	0.69	176.75
C11	79.42	99.86	79.56	99.73	79.47	99.82	79.65	99.64
C12	18.84	159.03	18.95	158.92	18.8	159.07	18.97	158.91
C15	9.06	168.58	9.41	168.24	9.13	168.51	9.34	168.31
C16	83.87	95.52	83.65	95.73	83.95	95.44	83.77	95.62
C17	62.69	116.21	62.17	116.72	62.67	116.23	62.08	116.8
C18	36.66	141.63	36.92	141.37	36.84	141.45	36.73	141.56
C19	48.74	129.83	48.76	129.81	48.84	129.74	48.72	129.85
C20	67.79	111.22	58.29	120.5	67.83	111.19	58.26	120.53
C21	30.94	147.21	31.53	146.64	30.95	147.21	31.74	146.44
C22	30.17	147.96	30.79	147.36	30.15	147.98	30.78	147.37
C23	64.02	114.91	63.14	115.77	64	114.93	63.03	115.87
C24	50.92	127.7	60.6	118.25	51.02	127.61	60.61	118.24
C27	51.28	127.35	51.33	127.3	51.66	126.98	51.46	127.18
C28	62.04	116.84	61.83	117.04	63.91	115.02	64.47	114.47
C29	32.08	146.1	32.03	146.15	31.87	146.3	31.86	146.31
C30	31.85	146.32	31.8	146.38	32.42	145.76	32.41	145.78
C31	65.36	113.6	65.4	113.56	65.13	113.82	65.09	113.86
C32	58.53	120.27	58.26	120.53	56.77	121.98	57.24	121.53
H35	25.91	5.91	25.95	5.87	25.93	5.89	25.9	5.92
H36	25	6.78	24.94	6.83	25	6.78	24.96	6.82
H37	23.9	7.83	23.93	7.8	23.95	7.79	23.97	7.77
Me	29.55	2.41	29.55	2.41	29.52	2.45	29.52	2.45

H41	26.66	5.19	26.64	5.21	26.65	5.2	26.67	5.18
H42	25.62	6.18	25.63	6.18	25.65	6.15	25.66	6.14
H43	24.14	7.6	24.57	7.19	24.17	7.57	24.58	7.18
H44	24.85	6.92	24.86	6.92	24.86	6.91	24.86	6.92
H45	24.71	7.06	24.23	7.52	24.73	7.04	24.27	7.48
H48	24.52	7.24	24.53	7.24	24.83	6.94	24.88	6.89
H49	25.03	6.75	25.02	6.76	24.83	6.94	24.83	6.95
H50	25.03	6.75	25.03	6.75	24.67	7.09	24.71	7.06

Table S3. xyz coordinates of conformers

SS_1:		angstroms	
atom	x	y	z
C1	4.7178230000	-4.1283200000	-1.0614620000
C2	4.6360420000	-2.6787290000	-0.7452750000
C3	5.4626700000	-1.8852180000	-0.0202760000
C4	4.8991580000	-0.5533100000	-0.0074080000
O5	5.2372110000	0.4645000000	0.5738210000
C6	3.6096910000	-0.6530710000	-0.8654530000
O7	3.5536060000	-2.0531820000	-1.2662410000
C8	2.3638770000	-0.2108110000	-0.0391010000
O9	1.4616510000	0.4412010000	-0.9958130000
C10	2.2040230000	0.7580420000	-2.0740580000
C11	3.4687020000	0.2246760000	-2.0571460000
C12	4.3733420000	0.3903900000	-3.1449990000
O13	5.4793280000	-0.0653860000	-3.3069090000
O14	3.8557840000	1.2339310000	-4.1785630000
C15	2.6131040000	1.7729690000	-4.1745600000
C16	1.7342300000	1.5612780000	-3.1388910000
C17	2.2999790000	2.5782390000	-5.3358480000
C18	3.1590360000	2.7810120000	-6.3603290000
C19	2.9338970000	3.5707240000	-7.5649860000
C20	1.7416540000	4.2823280000	-7.8145610000
C21	1.5812710000	5.0161840000	-8.9784820000
C22	2.6219760000	5.0548520000	-9.9301170000
C23	3.8037190000	4.3604100000	-9.7001090000
C24	3.9582080000	3.6252320000	-8.5263350000
O25	2.3604200000	5.8093080000	-11.0459570000
O26	0.4212140000	5.6954780000	-9.1953820000
C27	1.6735030000	-1.2963910000	0.7280000000
C28	0.7913100000	-2.1763430000	0.0903730000
C29	0.2223700000	-3.2283780000	0.7958400000
C30	0.5497940000	-3.4129440000	2.1531830000
C31	1.4302530000	-2.5448590000	2.7866670000
C32	1.9897930000	-1.4807900000	2.0744580000
O33	-0.0618750000	-4.4877870000	2.7570720000
O34	-0.6400840000	-4.0771720000	0.1683490000
H35	2.7247450000	0.5803380000	0.6285180000
H36	1.3075830000	3.0183320000	-5.3343110000
H37	4.1332800000	2.3029840000	-6.2895400000
H38	5.5995230000	-4.5804660000	-0.6046510000
H39	3.8164220000	-4.6353590000	-0.7010980000
H40	4.7602020000	-4.2669910000	-2.1469510000
H41	6.3744840000	-2.1896200000	0.4709430000
H42	0.7432840000	1.9934560000	-3.1420920000
H43	0.9197920000	4.2799770000	-7.1073430000
H44	4.6017920000	4.3951740000	-10.4379960000
H45	4.8842420000	3.0856510000	-8.3532890000
H46	3.1176250000	5.7911940000	-11.6445090000
H47	0.4941740000	6.1465180000	-10.0495810000
H48	0.5407750000	-2.0496030000	-0.9560660000

H49	1.6749110000	-2.6949020000	3.8356140000
H50	2.6797620000	-0.8040660000	2.5692080000
H51	0.1908170000	-4.5302570000	3.6875090000
H52	-0.9367960000	-4.7307830000	0.8188950000

SS_2:

angstroms

atom	x	y	z
C1	4.7032950000	-4.1129840000	-1.2094380000
C2	4.6075660000	-2.6720520000	-0.8594010000
C3	5.4458160000	-1.8781990000	-0.1484880000
C4	4.8598590000	-0.5576810000	-0.0859130000
O5	5.2007130000	0.4537980000	0.5045880000
C6	3.5409290000	-0.6642300000	-0.8968340000
O7	3.4953650000	-2.0558300000	-1.3268510000
C8	2.3215640000	-0.2644540000	-0.0124120000
O9	1.3655480000	0.3798310000	-0.9213130000
C10	2.0603370000	0.7409660000	-2.0174030000
C11	3.3365050000	0.2375050000	-2.0612090000
C12	4.1987370000	0.4560340000	-3.1744740000
O13	5.3080770000	0.0315850000	-3.3873850000
O14	3.6264480000	1.3181840000	-4.1646970000
C15	2.3736250000	1.8268320000	-4.0992190000
C16	1.5357380000	1.5622120000	-3.0416500000
C17	1.9979430000	2.6651150000	-5.2170000000
C18	2.8058050000	2.9388420000	-6.2661880000
C19	2.4976790000	3.7768860000	-7.4212300000
C20	3.4720340000	3.9009980000	-8.4323560000
C21	3.2487810000	4.6795990000	-9.5596750000
C22	2.0261980000	5.3609160000	-9.6951330000
C23	1.0522440000	5.2513850000	-8.7042430000
C24	1.2816420000	4.4682830000	-7.5771300000
O25	1.8958540000	6.1063890000	-10.8402850000
O26	4.2119820000	4.7748540000	-10.5170890000
C27	1.6909160000	-1.3744170000	0.7705910000
C28	0.8172630000	-2.2807080000	0.1580670000
C29	0.3048770000	-3.3518920000	0.8779630000
C30	0.6806430000	-3.5290080000	2.2236680000
C31	1.5525420000	-2.6348790000	2.8320540000
C32	2.0549980000	-1.5519480000	2.1059730000
O33	0.1226250000	-4.6241930000	2.8425560000
O34	-0.5494180000	-4.2265880000	0.2757520000
H35	2.6930140000	0.5262780000	0.6499450000
H36	0.9939410000	3.0741280000	-5.1583800000
H37	3.7960790000	2.4893520000	-6.2596290000
H38	5.6079850000	-4.5587020000	-0.7931400000
H39	3.8238270000	-4.6435230000	-0.8292420000
H40	4.7103520000	-4.2281800000	-2.2985020000
H41	6.3802520000	-2.1750490000	0.3021550000
H42	0.5361540000	1.9715680000	-2.9973350000
H43	4.4235860000	3.3853330000	-8.3496230000
H44	0.1115650000	5.7850390000	-8.8190810000
H45	0.5094590000	4.4028980000	-6.8188510000

H46	1.0361190000	6.5447390000	-10.8565220000
H47	3.8814460000	5.3558400000	-11.2181030000
H48	0.5304380000	-2.1602050000	-0.8797940000
H49	1.8349320000	-2.7794690000	3.8721860000
H50	2.7385100000	-0.8547340000	2.5807250000
H51	0.4090280000	-4.6638350000	3.7632460000
H52	-0.8027000000	-4.8920750000	0.9325710000

SS_3:

angstroms

atom	x	y	z
C1	4.7348630000	-4.0640000000	-1.1125540000
C2	4.5564510000	-2.6278190000	-0.7769410000
C3	5.2991920000	-1.8084030000	0.0075260000
C4	4.6666550000	-0.5086890000	0.0124630000
O5	4.9173640000	0.5147760000	0.6263500000
C6	3.4358620000	-0.6562870000	-0.9240380000
O7	3.4718140000	-2.0497250000	-1.3468750000
C8	2.1168270000	-0.2847850000	-0.1758970000
O9	1.2811250000	0.4189430000	-1.1524450000
C10	2.0795150000	0.7654640000	-2.1793300000
C11	3.3446070000	0.2405070000	-2.1067570000
C12	4.3054890000	0.4377220000	-3.1403530000
O13	5.4244710000	-0.0013080000	-3.2494200000
O14	3.8329060000	1.2901900000	-4.1885540000
C15	2.5863330000	1.8162920000	-4.2396430000
C16	1.6590650000	1.5849640000	-3.2517040000
C17	2.3164140000	2.6239530000	-5.4083680000
C18	3.2106450000	2.8411530000	-6.3988820000
C19	3.0086210000	3.6256290000	-7.6109820000
C20	1.8040320000	4.3004090000	-7.9016380000
C21	1.6608060000	5.0262820000	-9.0725720000
C22	2.7315080000	5.0945260000	-9.9888620000
C23	3.9256880000	4.4374390000	-9.7174730000
C24	4.0626760000	3.7091580000	-8.5370000000
O25	2.4840840000	5.8379050000	-11.1153670000
O26	0.4885020000	5.6686610000	-9.3315490000
C27	1.3869780000	-1.4430060000	0.4394650000
C28	1.7984620000	-1.8938770000	1.6996870000
C29	1.2121710000	-3.0187670000	2.2685720000
C30	0.1938380000	-3.6941840000	1.5724250000
C31	-0.2197430000	-3.2427540000	0.3242570000
C32	0.3808820000	-2.1197610000	-0.2482770000
O33	-0.3270220000	-4.7904830000	2.2228880000
O34	1.6233970000	-3.4495490000	3.4937660000
H35	2.4005010000	0.4608150000	0.5755320000
H36	1.3188360000	3.0502260000	-5.4471920000
H37	4.1899080000	2.3801420000	-6.2937630000
H38	3.8499520000	-4.6281090000	-0.7992340000
H39	4.8329620000	-4.1794110000	-2.1971510000
H40	5.6200250000	-4.4732000000	-0.6233640000
H41	6.1958500000	-2.0781810000	0.5444810000

H42	0.6645570000	2.0060590000	-3.3012960000
H43	0.9590850000	4.2736300000	-7.2230910000
H44	4.7464950000	4.4951610000	-10.4283440000
H45	4.9979250000	3.1975900000	-8.3316330000
H46	3.2605860000	5.8416860000	-11.6888690000
H47	0.5770340000	6.1198180000	-10.1842730000
H48	2.5807670000	-1.3824130000	2.2520690000
H49	-1.0095990000	-3.7723300000	-0.2035430000
H50	0.0669900000	-1.7694200000	-1.2242530000
H51	-1.0451970000	-5.1706550000	1.7025800000
H52	1.0877190000	-4.2210450000	3.7310040000

SS_4:

angstroms

atom	x	y	z
C1	4.6202340000	-4.0964090000	-1.2957340000
C2	4.4632580000	-2.6650890000	-0.9295690000
C3	5.2449350000	-1.8596890000	-0.1690970000
C4	4.6144150000	-0.5604810000	-0.1078160000
O5	4.8950310000	0.4497700000	0.5151980000
C6	3.3378330000	-0.6913140000	-0.9821140000
O7	3.3529840000	-2.0756240000	-1.4354420000
C8	2.0615040000	-0.3426770000	-0.1558370000
O9	1.1568740000	0.3444600000	-1.0816100000
C10	1.8984650000	0.7314570000	-2.1380370000
C11	3.1753250000	0.2304000000	-2.1374750000
C12	4.0836800000	0.4730680000	-3.2085180000
O13	5.2021970000	0.0550640000	-3.3820600000
O14	3.5510070000	1.3511880000	-4.2061970000
C15	2.2960200000	1.8581850000	-4.1841470000
C16	1.4160550000	1.5740730000	-3.1663110000
C17	1.9667890000	2.7121160000	-5.3054320000
C18	2.8166260000	2.9908850000	-6.3200230000
C19	2.5605060000	3.8383830000	-7.4811010000
C20	3.5670300000	3.9465240000	-8.4622300000
C21	3.3915520000	4.7305330000	-9.5943630000
C22	2.1862270000	5.4347530000	-9.7651490000
C23	1.1818190000	5.3426310000	-8.8035040000
C24	1.3639270000	4.5534230000	-7.6721820000
O25	2.1031320000	6.1839170000	-10.9121720000
O26	4.3843710000	4.8091600000	-10.5228780000
C27	1.4000310000	-1.5133080000	0.5105010000
C28	1.9165890000	-1.9526820000	1.7361140000
C29	1.4051850000	-3.0924740000	2.3460640000
C30	0.3552170000	-3.7947630000	1.7274770000
C31	-0.1631550000	-3.3541860000	0.5148660000
C32	0.3632570000	-2.2163510000	-0.1005880000
O33	-0.0849140000	-4.9052080000	2.4125580000
O34	1.9208120000	-3.5121200000	3.5353440000
H35	2.3800920000	0.4100720000	0.5747850000
H36	0.9624210000	3.1239330000	-5.2827420000
H37	3.8029680000	2.5347940000	-6.2784030000

H38	4.6610490000	-4.1964650000	-2.3854860000
H39	5.5284610000	-4.5147420000	-0.8593810000
H40	3.7513020000	-4.6631510000	-0.9445550000
H41	6.1657310000	-2.1379260000	0.3197490000
H42	0.4147540000	1.9817080000	-3.1557740000
H43	4.5059960000	3.4133200000	-8.3522450000
H44	0.2552280000	5.8945710000	-8.9436150000
H45	0.5695840000	4.5033860000	-6.9361400000
H46	1.2537720000	6.6407780000	-10.9508430000
H47	4.0858770000	5.3985790000	-11.2312100000
H48	2.7253920000	-1.4210990000	2.2279910000
H49	-0.9763910000	-3.9049380000	0.0476230000
H50	-0.0313640000	-1.8752500000	-1.0502650000
H51	-0.8259380000	-5.3083670000	1.9441570000
H52	1.4284110000	-4.2999750000	3.8096520000

SR_1:

angstroms

atom	x	y	z
C1	2.6679740000	-5.0564050000	-0.1738160000
C2	2.4077510000	-3.5935870000	-0.1570100000
C3	1.9500360000	-2.7690190000	-1.1285120000
C4	1.8228500000	-1.4437710000	-0.5604180000
O5	1.4224990000	-0.3955180000	-1.0352410000
C6	2.2997370000	-1.5903000000	0.9131650000
O7	2.6553720000	-2.9931580000	1.0362680000
C8	3.5357610000	-0.7282300000	1.3539130000
O9	2.9879230000	0.3451090000	2.1878180000
C10	1.7370550000	-0.0155040000	2.5373480000
C11	1.2823660000	-1.1414950000	1.9035160000
C12	-0.0335820000	-1.6454850000	2.1069790000
O13	-0.5859030000	-2.5889830000	1.5930270000
O14	-0.7684560000	-0.9093820000	3.0862540000
C15	-0.3050910000	0.1964050000	3.7176400000
C16	0.9523450000	0.6953440000	3.4747850000
C17	-1.2327210000	0.7838630000	4.6603360000
C18	-2.4683900000	0.2886780000	4.8979190000
C19	-3.4660220000	0.7966960000	5.8310280000
C20	-3.2444410000	1.9062260000	6.6731960000
C21	-4.2284310000	2.3451830000	7.5439060000
C22	-5.4696020000	1.6762990000	7.5915310000
C23	-5.7044280000	0.5807400000	6.7691620000
C24	-4.7094640000	0.1442750000	5.8965840000
O25	-6.3723600000	2.1910890000	8.4878480000
O26	-3.9906860000	3.4172950000	8.3491100000
C27	4.4257060000	-0.1782820000	0.2787870000
C28	4.0775720000	0.9775600000	-0.4302240000
C29	4.8976310000	1.4414950000	-1.4506580000
C30	6.0712070000	0.7353910000	-1.7774880000
C31	6.4154290000	-0.4158400000	-1.0801360000
C32	5.5945310000	-0.8695830000	-0.0452130000
O33	6.8107270000	1.2793020000	-2.8028260000

O34	4.5596140000	2.5731950000	-2.1276910000
H35	4.1159320000	-1.3762800000	2.0206970000
H36	-0.8658920000	1.6646700000	5.1780250000
H37	-2.7635710000	-0.5918050000	4.3329140000
H38	1.9946660000	-5.5537250000	0.5326630000
H39	2.5100730000	-5.4690200000	-1.1714210000
H40	3.6938090000	-5.2626580000	0.1490860000
H41	1.6896270000	-3.0518930000	-2.1370190000
H42	1.3166640000	1.5794560000	3.9792020000
H43	-2.3033870000	2.4435790000	6.6672610000
H44	-6.6634900000	0.0699780000	6.8119220000
H45	-4.8990510000	-0.7120560000	5.2567860000
H46	-7.1936510000	1.6845200000	8.4603740000
H47	-4.7853270000	3.5731890000	8.8808150000
H48	3.1602640000	1.5083030000	-0.2098280000
H49	7.3280340000	-0.9492160000	-1.3356240000
H50	5.8696440000	-1.7636490000	0.5063940000
H51	7.5705320000	0.7163990000	-2.9948150000
H52	5.2293920000	2.7211280000	-2.8115580000

SR_2:

angstroms

atom	x	y	z
C1	2.4480280000	-4.9773160000	-0.3032320000
C2	2.2496040000	-3.5062200000	-0.2367320000
C3	1.7988640000	-2.6351010000	-1.1702670000
C4	1.7497690000	-1.3222410000	-0.5631560000
O5	1.3821390000	-0.2441010000	-0.9958900000
C6	2.2707430000	-1.5304870000	0.8879300000
O7	2.5588160000	-2.9516640000	0.9643840000
C8	3.5670690000	-0.7455120000	1.2987470000
O9	3.1056730000	0.3455100000	2.1614930000
C10	1.8515570000	0.0440670000	2.5532750000
C11	1.3167300000	-1.0499400000	1.9262010000
C12	-0.0171190000	-1.4852470000	2.1716130000
O13	-0.6372710000	-2.3901280000	1.6666940000
O14	-0.6768310000	-0.7243950000	3.1873760000
C15	-0.1332920000	0.3489440000	3.8096310000
C16	1.1391140000	0.7838660000	3.5249260000
C17	-0.9892620000	0.9799620000	4.7913170000
C18	-2.2386330000	0.5554610000	5.0866130000
C19	-3.1595070000	1.1245740000	6.0654460000
C20	-4.4326160000	0.5346270000	6.2014310000
C21	-5.3598630000	1.0204750000	7.1127990000
C22	-5.0219150000	2.1231030000	7.9178510000
C23	-3.7676160000	2.7180590000	7.7975310000
C24	-2.8429420000	2.2263950000	6.8812830000
O25	-6.0007640000	2.5314500000	8.7895320000
O26	-6.5804860000	0.4262660000	7.2173250000
C27	4.4561530000	-0.2315980000	0.2051720000
C28	4.1460700000	0.9438250000	-0.4897100000
C29	4.9650740000	1.3774380000	-1.5241850000

C30	6.0990890000	0.6219220000	-1.8792930000
C31	6.4053230000	-0.5484290000	-1.1963000000
C32	5.5859870000	-0.9721340000	-0.1473940000
O33	6.8413380000	1.1396630000	-2.9160750000
O34	4.6646910000	2.5277670000	-2.1874470000
H35	4.1287670000	-1.4345200000	1.9398810000
H36	-0.5512640000	1.8379720000	5.2918610000
H37	-2.6093110000	-0.3077300000	4.5393890000
H38	3.4752300000	-5.2335810000	-0.0231600000
H39	1.7800330000	-5.4681110000	0.4127390000
H40	2.2392630000	-5.3539380000	-1.3057150000
H41	1.4964150000	-2.8776200000	-2.1775740000
H42	1.5675110000	1.6425230000	4.0228540000
H43	-4.7155170000	-0.3169300000	5.5906220000
H44	-3.5175550000	3.5690660000	8.4267320000
H45	-1.8731130000	2.7046680000	6.8077140000
H46	-5.6962760000	3.2917260000	9.3001000000
H47	-7.0869630000	0.8986790000	7.8944390000
H48	3.2577560000	1.5131850000	-0.2481070000
H49	7.2876400000	-1.1203580000	-1.4737930000
H50	5.8320850000	-1.8813750000	0.3929670000
H51	7.5729790000	0.5463980000	-3.1252880000
H52	5.3267790000	2.6503530000	-2.8837220000

SR_3:

angstroms

atom	x	y	z
C1	2.8089620000	-4.8285400000	-0.1983590000
C2	2.4496050000	-3.3871020000	-0.1804130000
C3	1.9081860000	-2.6016950000	-1.1421260000
C4	1.7145840000	-1.2829170000	-0.5787610000
O5	1.2367920000	-0.2643240000	-1.0478690000
C6	2.2434360000	-1.3869390000	0.8807050000
O7	2.6920710000	-2.7627640000	1.0014610000
C8	3.4348430000	-0.4424260000	1.2772300000
O9	2.8499420000	0.5876750000	2.1395490000
C10	1.6335960000	0.1527730000	2.5222460000
C11	1.2286500000	-0.9978560000	1.8992790000
C12	-0.0530430000	-1.5731220000	2.1321880000
O13	-0.5640180000	-2.5457940000	1.6300620000
O14	-0.8065620000	-0.8768570000	3.1272450000
C15	-0.3906460000	0.2534110000	3.7470540000
C16	0.8327340000	0.8190020000	3.4782040000
C17	-1.3298960000	0.7986900000	4.7029260000
C18	-2.5395160000	0.2525950000	4.9613420000
C19	-3.5420030000	0.7306740000	5.9055970000
C20	-3.3446310000	1.8504880000	6.7403120000
C21	-4.3313230000	2.2645950000	7.6199610000
C22	-5.5516460000	1.5599110000	7.6846230000
C23	-5.7622430000	0.4533640000	6.8704330000
C24	-4.7643220000	0.0415510000	5.9889380000
O25	-6.4593800000	2.0527720000	8.5880190000

O26	-4.1155620000	3.3474020000	8.4170810000
C27	4.2322760000	0.1776970000	0.1673050000
C28	5.4227000000	-0.4436270000	-0.2294460000
C29	6.1620570000	0.0611430000	-1.2932740000
C30	5.7095860000	1.2122120000	-1.9600910000
C31	4.5295390000	1.8339490000	-1.5639990000
C32	3.7843560000	1.3159190000	-0.5052580000
O33	6.5149490000	1.6441540000	-2.9892390000
O34	7.3183440000	-0.5580220000	-1.6681750000
H35	4.0877760000	-1.0496570000	1.9142810000
H36	-0.9955270000	1.6978110000	5.2110230000
H37	-2.8074070000	-0.6430840000	4.4058690000
H38	2.6424460000	-5.2591120000	-1.1868960000
H39	3.8584550000	-4.9600260000	0.0852750000
H40	2.1992600000	-5.3650500000	0.5366950000
H41	1.6394740000	-2.9090590000	-2.1415160000
H42	1.1584740000	1.7230260000	3.9735640000
H43	-2.4199210000	2.4153750000	6.7235150000
H44	-6.7051940000	-0.0854320000	6.9264600000
H45	-4.9360140000	-0.8233500000	5.3556160000
H46	-7.2644070000	1.5202150000	8.5745110000
H47	-4.9073850000	3.4821540000	8.9586620000
H48	5.7923250000	-1.3284120000	0.2799080000
H49	4.1872790000	2.7209410000	-2.0918930000
H50	2.8499340000	1.7788020000	-0.2155140000
H51	6.1148990000	2.4101430000	-3.4186010000
H52	7.6859490000	-0.0646110000	-2.4163280000

SR_4:

angstroms

atom	x	y	z
C1	2.6730690000	-4.7834830000	-0.1850930000
C2	2.3637700000	-3.3307200000	-0.1536250000
C3	1.8236900000	-2.5240220000	-1.0982230000
C4	1.6942670000	-1.2005300000	-0.5283690000
O5	1.2407530000	-0.1639150000	-0.9819070000
C6	2.2654970000	-1.3257660000	0.9131870000
O7	2.6615750000	-2.7182260000	1.0218770000
C8	3.5090950000	-0.4298970000	1.2604910000
O9	2.9986040000	0.6289780000	2.1345830000
C10	1.7777000000	0.2485390000	2.5612620000
C11	1.3044020000	-0.8891840000	1.9639090000
C12	0.0062950000	-1.4055520000	2.2427620000
O13	-0.5619830000	-2.3581150000	1.7657570000
O14	-0.6848140000	-0.6687970000	3.2558170000
C15	-0.2005980000	0.4476120000	3.8496710000
C16	1.0372190000	0.9568290000	3.5358770000
C17	-1.0817230000	1.0444200000	4.8304120000
C18	-2.3064350000	0.5621470000	5.1401850000
C19	-3.2490370000	1.0995230000	6.1169350000
C20	-4.5098840000	0.4800160000	6.2338380000
C21	-5.4582280000	0.9364250000	7.1388420000

C22	-5.1539570000	2.0372930000	7.9590800000
C23	-3.9108570000	2.6596780000	7.8596360000
C24	-2.9653290000	2.1985170000	6.9487190000
O25	-6.1506230000	2.4154880000	8.8240330000
O26	-6.6669140000	0.3151510000	7.2227390000
C27	4.2865850000	0.1462500000	0.1127660000
C28	5.4058700000	-0.5531170000	-0.3552170000
C29	6.1130780000	-0.0937710000	-1.4605540000
C30	5.7022590000	1.0894310000	-2.0976910000
C31	4.5943210000	1.7888110000	-1.6305400000
C32	3.8796720000	1.3162410000	-0.5299700000
O33	6.4726790000	1.4700580000	-3.1729470000
O34	7.1991470000	-0.7879240000	-1.9063590000
H35	4.1599180000	-1.0596370000	1.8776300000
H36	-0.6870290000	1.9327090000	5.3142480000
H37	-2.6388390000	-0.3238970000	4.6049750000
H38	2.0710550000	-5.3012600000	0.5693530000
H39	2.4592470000	-5.2058780000	-1.1680410000
H40	3.7265040000	-4.9507860000	0.0627470000
H41	1.5160890000	-2.8186960000	-2.0899200000
H42	1.4177770000	1.8504240000	4.0107280000
H43	-4.7673900000	-0.3710090000	5.6112360000
H44	-3.6850500000	3.5075120000	8.5023270000
H45	-2.0035290000	2.6954550000	6.8955300000
H46	-5.8661000000	3.1739500000	9.3487060000
H47	-7.1902010000	0.7682010000	7.9003210000
H48	5.7422020000	-1.4650760000	0.1284990000
H49	4.2829860000	2.7004460000	-2.1351520000
H50	2.9991640000	1.8409580000	-0.1833560000
H51	6.0965830000	2.2587710000	-3.5824240000
H52	7.5523030000	-0.3174050000	-2.6760060000

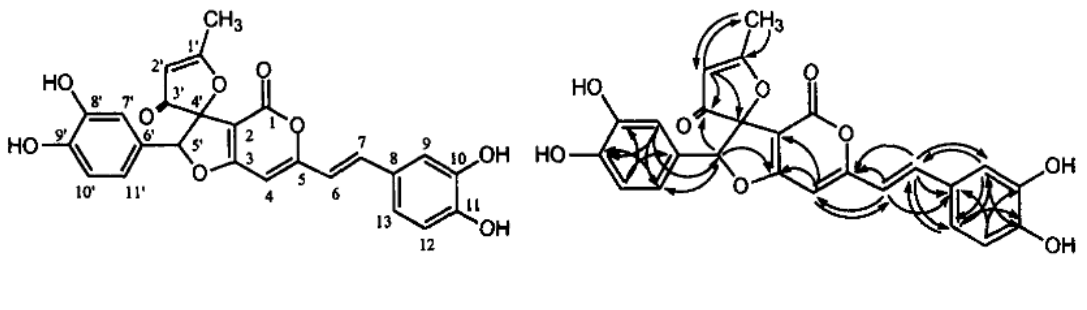

Figure S6. Literature structure of compound **2**[1]

Literature Inoscavin A: [O.]D=0 (c=9.0, CH_3OH), UV ~.max nm (g) in MeOH : 262 (11,822), 389 (11,568); IR Vmax (KBr) : 3430, 1700, 1695, 1593, 1549, 1384, 1276, 1127 cm q ; HRFAB-MS ; m/z found 463.1045, C25H1809 requires 463.1029 ; 1H NMR (600MHz, CD_3OD): 8 1.98 (s, Me-I '), 5.57 (s, H-2'), 5.65 (s, H-

5'), 6.50 (s, n-4), 6.59 (dd, ./=8.1, 1.6, H-11'), 6.71 (d, .]=1.6, H-7'), 6.72 (d, J=15.9, H-6), 6.75 (d, ./--8.1, H-10'), 6.79 (d, J=8.2, H-12), 7.00 (dd, J=8.2, 1.8, H-13), 7.08 (d, ./=1.8, H-9), 7.44 (d, J=15.9, H-7). 13C NMR (150 MHz, CD3OD): 8 20.0 (Me-I), 94.5 (C-4'), 95.5 (C-4), 95.9 (C-5'), 99.5 (C-2), 105.1 (C-2'), 115.0 (C-9), 115.1 (C-7'), 115.5 (C-10'), 115.8 (C-12), 116.6 (C-6), 120.2 (C-11'), 122.7 (C-13), 123.2 (C-6'), 128.5 (C-8), 139.9 (C-7), 146.3 (C-8'), 147.0 (C-10), 147.8 (C-9'), 149.5 (C-11), 160.6 (C-1), 167.0 (C-5), 176.8 (C-3), 192.9 (C-l'), 203.1 (C-3').

^{13}C NMR (201 MHz, METHANOL-d_4) δ = 203.3 (C-3'), 193.1 (C-1'), 177.0 (C-3), 167.2 (C-5), 160.8 (C-1), 149.6 (C-11), 148.0 (C-9'), 147.1 (C-10), 146.4 (C-8'), 140.0 (C-7), 128.6 (C-8), 123.3 (C-6'), 122.8 (C-13), 120.4 (C-11'), 116.8 (C-6, C-10'), 116.1 (C-12), 115.6 (C-7'), 115.2 (C-9), 105.3 (C-2'), 99.6 (C-2), 96.0 (C-5'), 95.7 (C-4), 94.4 (C-4'), 16.8 (Me)

^{1}H NMR (800 MHz, METHANOL-d_4) δ = 7.47 (1H, d, J = 15.9 Hz, H-7), 7.10 (1H, d, J = 2.0 Hz, H-9), 7.02 (1H, dd, J = 8.2 Hz, J = 2.3 Hz, H-13), 6.82 (1H, d, J = 8.3 Hz, H-10'), 6.78 (2H, d, J = 8.3 Hz, H-12), 6.75 (1H, d, J = 15.9 Hz, H-6), 6.73 (1H, d, J = 2.2 Hz, H-7'), 6.61 (1H, dd, J = 8.3 Hz, J = 2.2 Hz, H-11'), 6.53 (1H, s, H-4), 5.68 (1H, s, H-5'), 5.60 (1H, s, H-2'), 2.00 (3H, s, Me),

^{13}C NMR (201 MHz, METHANOL-d_4) δ = 203.3 (C-3'c), 193.1 (C-1'c), 177.0 (C-3c), 167.2 (C-5c), 160.8 (C-1c), 148.2 (C-11c), 148.0 (C-9'c), 147.1 (C-10c), 146.4 (C-8'c), 141.5 (C-7c), 128.6 (C-8c), 123.7 (C-13c), 123.3 (C-6'c), 120.4 (C-7'c), 118.6 (C-6c), 117.5 (C-9c), 116.4 (C-12c), 116.0 (C-10'c), 115.6 (C-11'c), 105.3 (C-2'c), 100.2 (C-2c), 97.9 (C-4c), 96.0 (C-5'c), 94.4 (C-4'), 16.7 (Mec)

^{1}H NMR (800 MHz, METHANOL-d_4) δ = 7.00 (1H, d, J = 2.0 Hz, H-9c), 6.88 (1H, dd, J = 8.2 Hz, J = 2.3 Hz, H-13c), 6.87 (1H, d, J = 12.7 Hz, H-7c), 6.78 (1H, d, J = 8.3 Hz, H-12c), 6.74 (2H, d, J = 8.3 Hz, H-10'c), 6.71 (1H, d, J = 2.2 Hz, H-7'c), 6.59 (1H, dd, J = 8.3 Hz, J = 2.2 Hz, H-11'), 6.51 (1H, s, H-4), 6.12 (1H, d, J = 12.7 Hz, H-6c), 5.67 (1H, s, H-5'c), 5.59 (1H, s, H-2'c), 1.99 (3H, s, Mec),

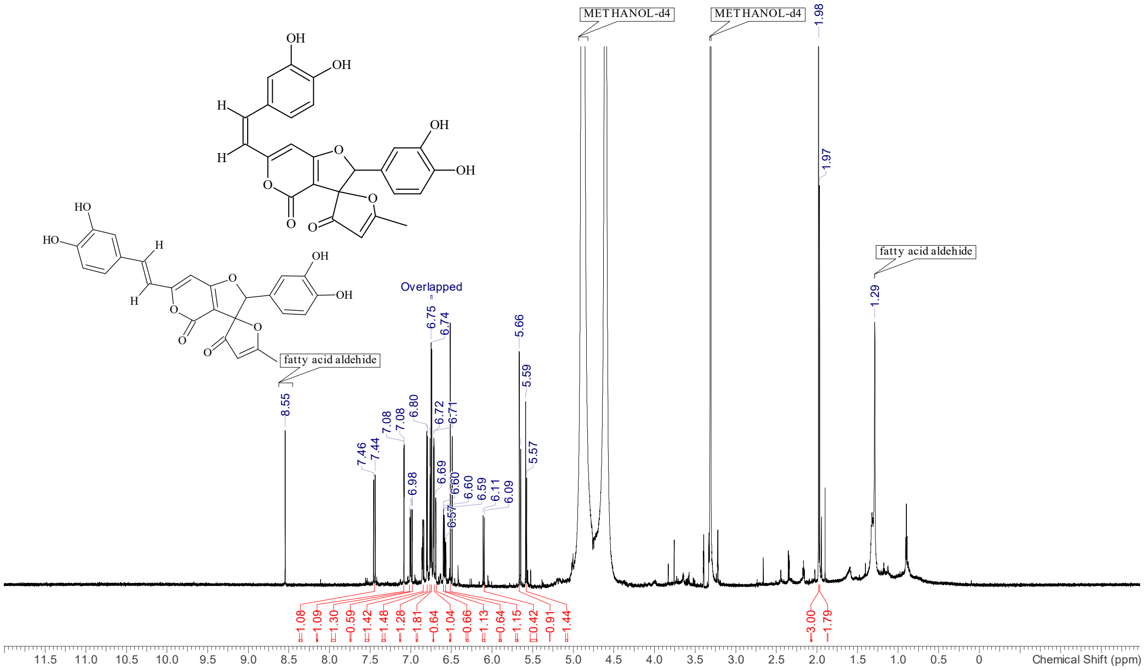

Figure S7. 800 MHz 1H NMR spectrum of mixture of compounds **2** and **3**

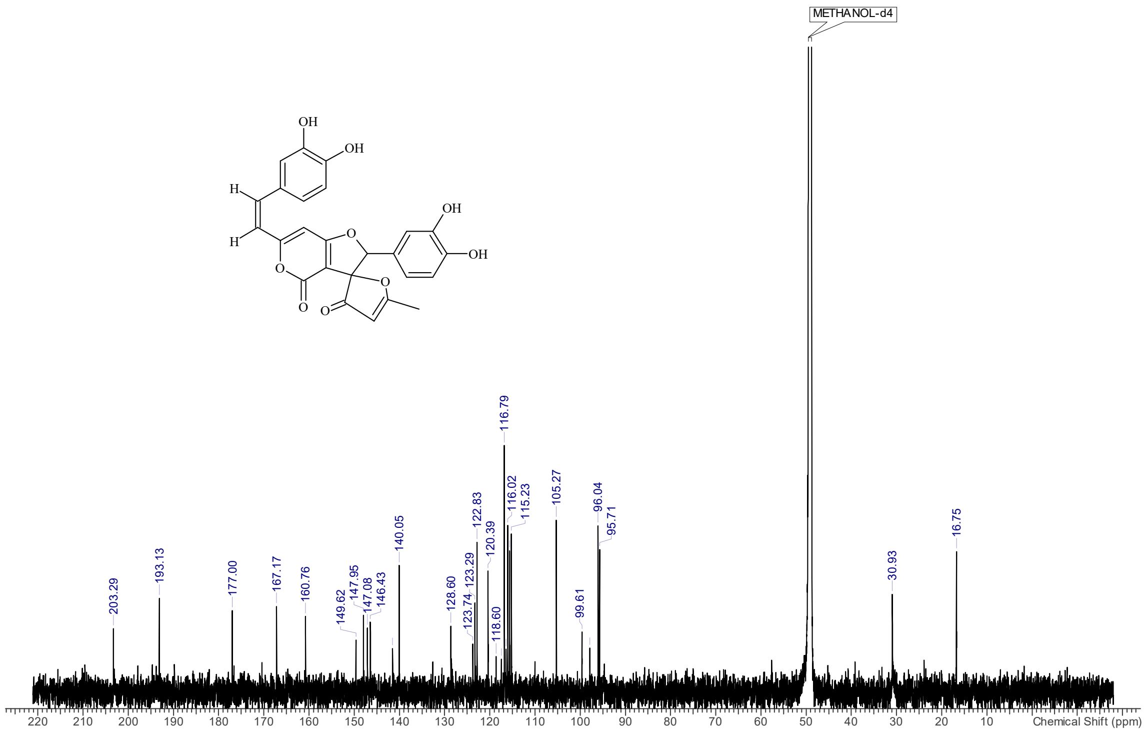

Figure S8. 800 MHz 13C NMR spectrum of mixture of compounds **2** and **3**

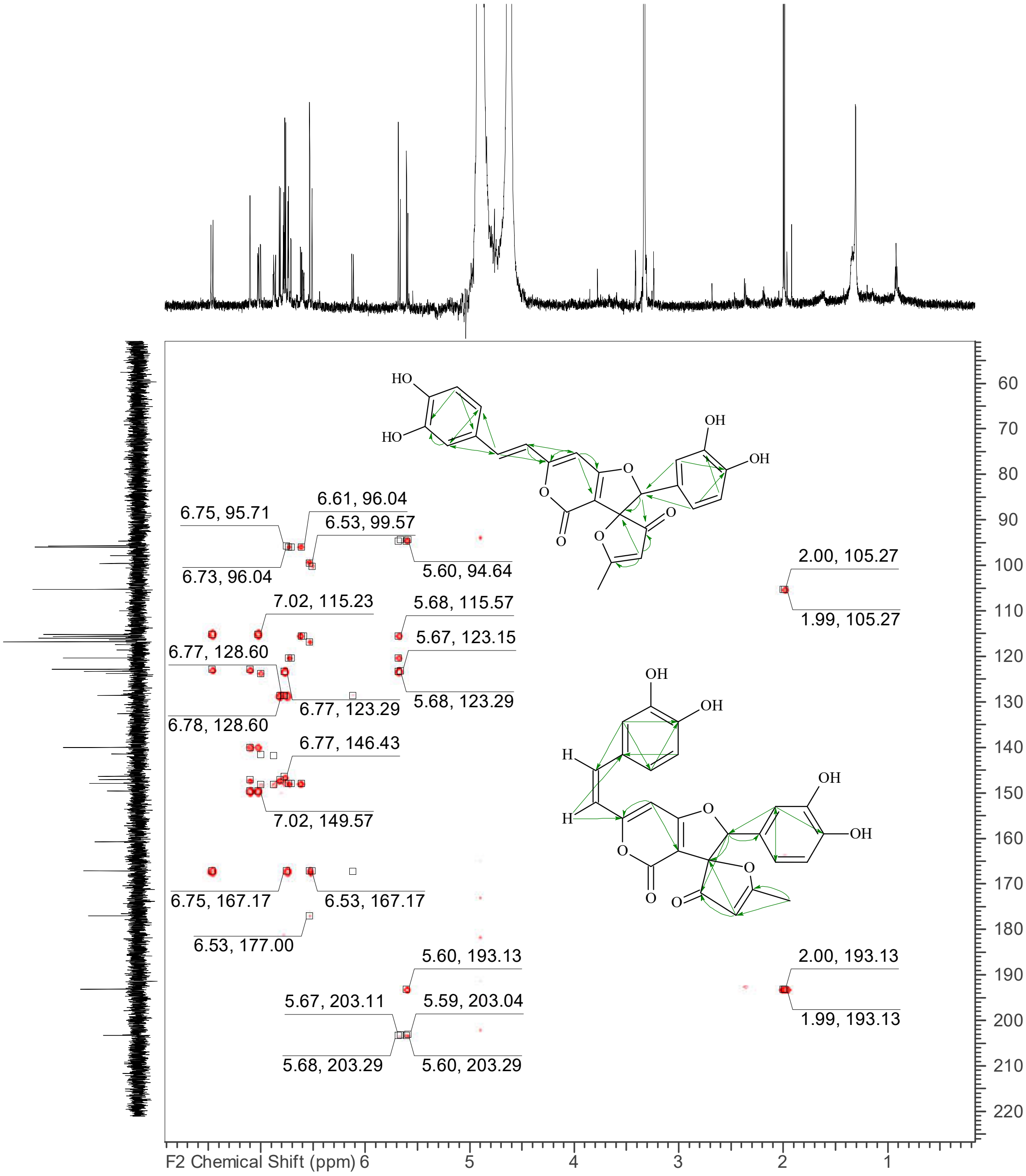

Figure S9. 800 MHz HMBC spectrum of mixture of compounds **2** and **3**

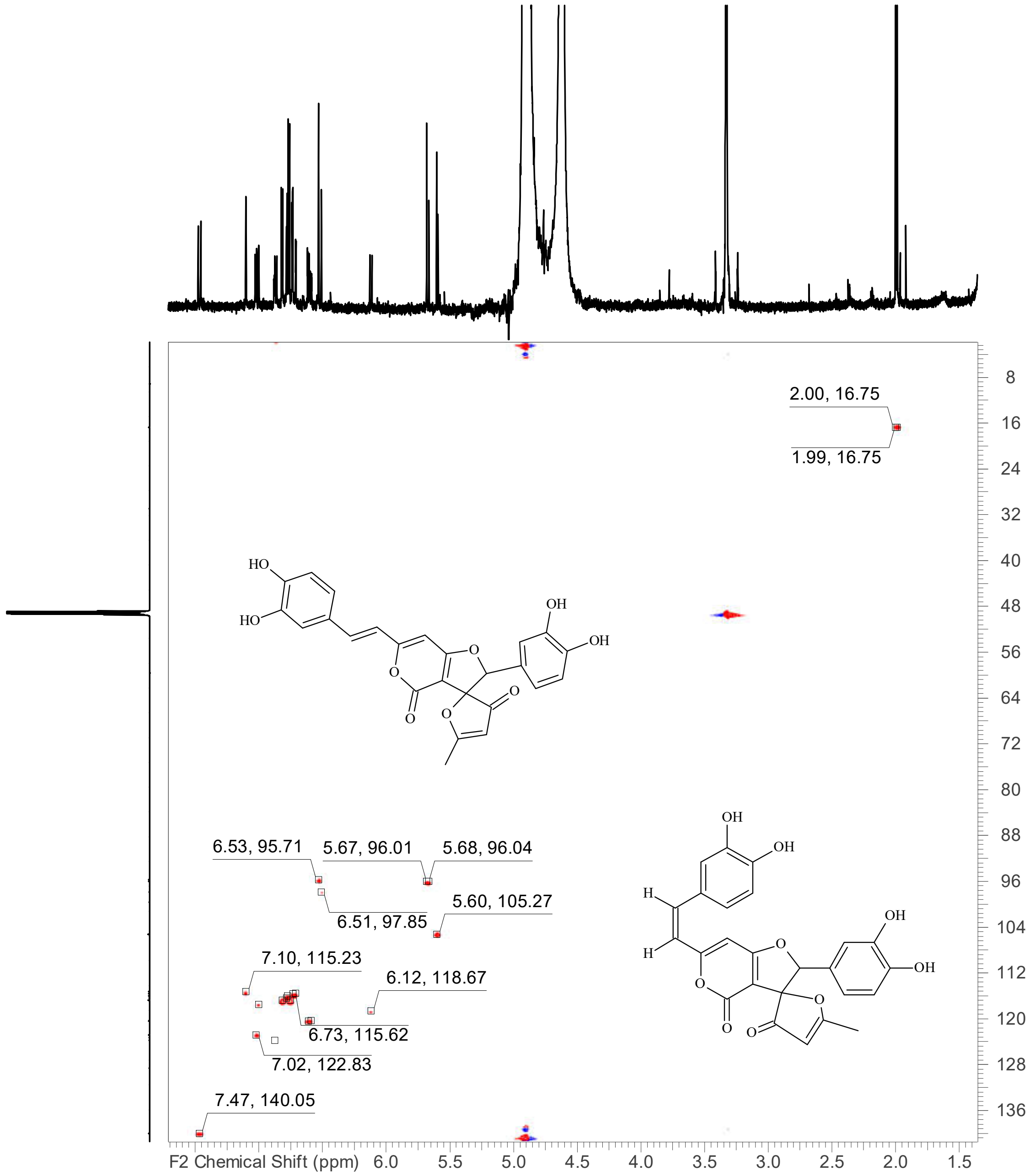

Figure S10. 800 MHz HSQC spectrum of mixture of compounds **2** and **3**

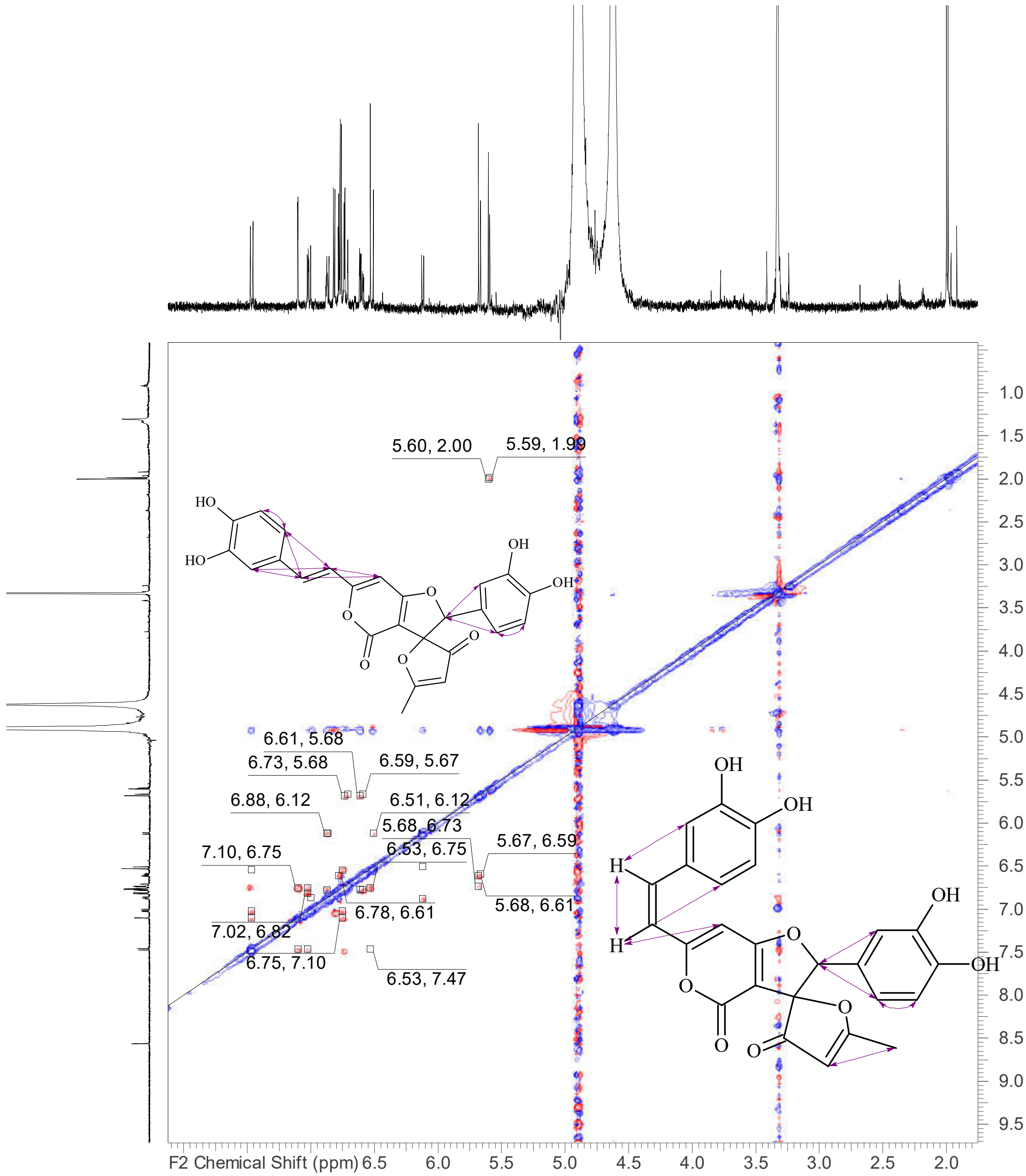

Figure S11. 800 MHz ROESY spectrum of mixture of compounds **2** and **3**

Spectra and spectral data on compounds 4 and 5

The sample is an equimolar mixture of 3,4-dihydroxy-benzaldehide and osmundacetone. NMR data are given for both.

HRMS: M+H=139.03857 (delta=-2.9 ppm; $C_7H_7O_3$). HR-ESI-MS-MS (CID=35%; rel. int. %): 111(100); 93(9).

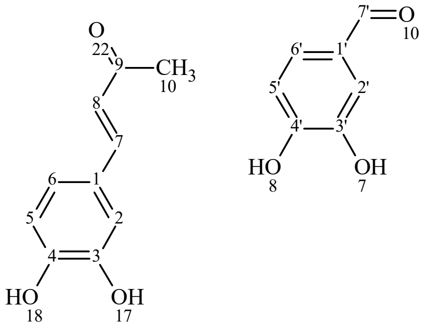

^{1}H NMR (800 MHz, METHANOL-d$_4$) δ = 7.55 (1H, d, $J_{7,8}$ = 16.1 Hz, H-7), 7.33 (1H, dd, J = 8.1 Hz, J = 2.0 Hz, M10), 7.10 (1H, d, $J_{2,6}$ = 2.2 Hz, H-2), 7.02 (1H, dd, J = 8.2 Hz, $J_{6,2}$ = 2.1 Hz, H-6), 6.81 (1H, d, $J_{5,5'}$ = 8.3 Hz, H-5), 6.58 (1H, d, $J_{8,7}$ = 16.1 Hz, H-8), 2.36 (3H, s, H-10)

^{1}H NMR (800 MHz, METHANOL-d$_4$) δ = 9.71 (1H, s, H-7'), 7.33 (1H, dd, $J_{6',6}$ = 8.1 Hz, $J_{6',2'}$ = 2.0 Hz, H-6'), 7.31 (1H, d, $J_{2',6'}$ = 2.0 Hz, H-2'), 6.93 (1H, d, $J_{5',5}$ = 8.1 Hz, H-5')

^{13}C NMR (201 MHz, METHANOL-d$_4$) δ = 201.7 (C-9), 150.2 (C-4), 147.1 (C-3), 147.0 (C-7), 127.9 (C-1), 124.9 (C-8), 123.7 (C-6), 116.7 (C-5), 115.4 (C-2), 27.2 (C-10)

^{13}C NMR (201 MHz, METHANOL-d$_4$) δ = 193.2 (C-7'), 153.9 (C-4'), 147.4 (C-3'), 131.0 (C-1'), 126.6 (C-6'), 116.4 (C-5'), 115.5 (C-2'),

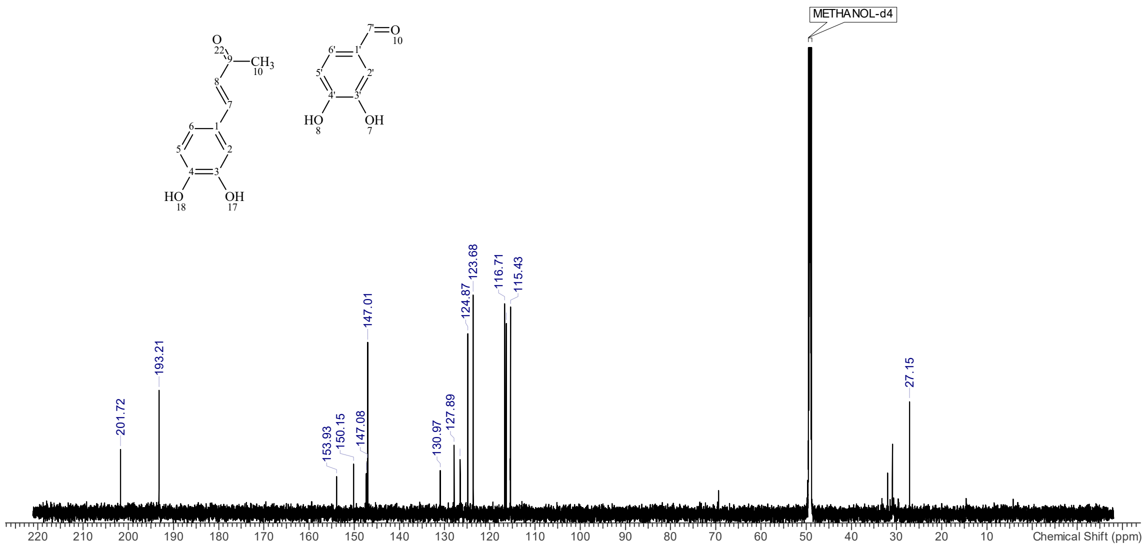

Figure S12. 200 MHz 13C NMR spectrum of mixture of compounds **4** and **5**

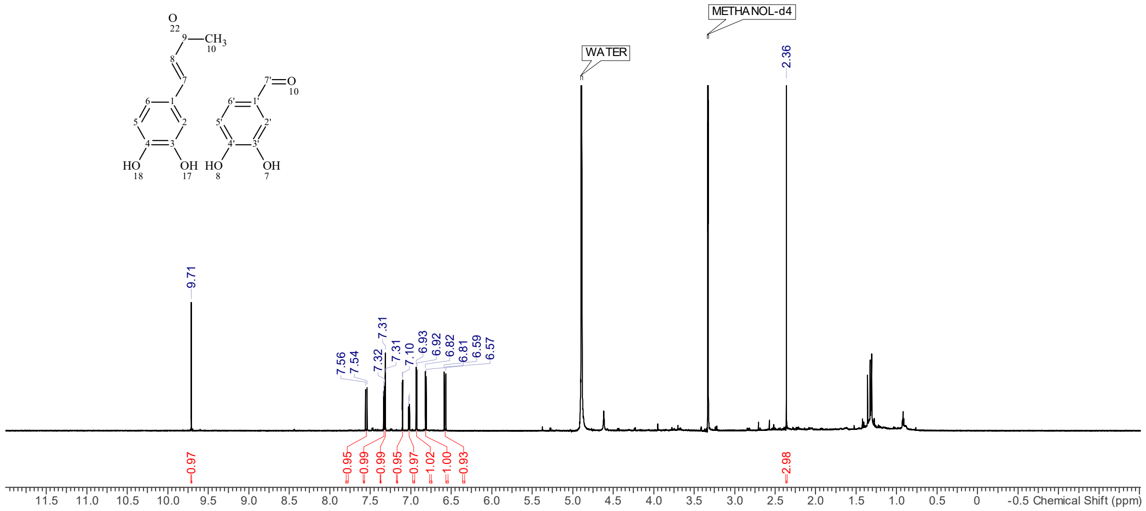

Figure S13. 800 MHz 1H NMR spectrum mixture of compounds **4** and **5**

Spectra and spectral data on compound 6

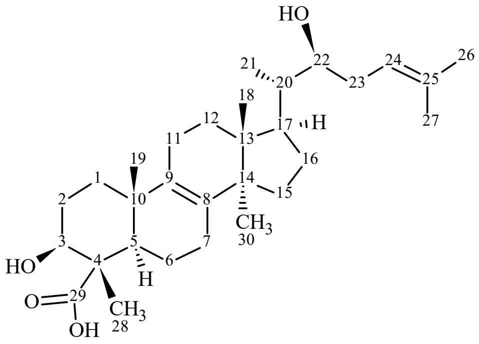

HRMS: M-H=471.34689 (delta=-2.3 ppm; $C_{30}H_{47}O_4$). HR-ESI-MS-MS (CID=35%; rel. int. %): 453(100); 425(58); 415(43); 339(8).

Known compound: senexdiolic acid. Although in our case NMR data were collected in MeOD, they are in good agreement with those riported in the literature in $CDCl_3$:MeOD mixture. [2–4]

^{13}C NMR (201 MHz, METHANOL-d$_4$) δ = 135.9 (C-9), 135.9 (C-8), 133.3 (C-25), 123.6 (C-24), 76.4 (C-3), 75.0 (C-22), 50.8 (C-14), 48.7 (C-17), 48.1 (C-5), 46.1 (C-13), 44.3 (C-20), 37.7 (C-10), 36.9 (C-1), 32.4 (C-12), 32.1 (C-15), 30.3 (C-23), 28.6 (C-16), 28.3 (C-2), 27.3 (C-7), 24.8 (C-30), 22.2 (C-11), 21.9 (C-6), 19.9 (C-19), 18.1 (C-27), 16.3 (C-18), 13.2 (C-21), 11.6 (C-28)

^{1}H NMR (800 MHz, METHANOL-d$_4$) δ = 5.26 (1H, m, H-24), 3.98 (1H, dd, J = 11.4 Hz, J = 5.0 Hz, H-3), 3.64 (1H, dt, J = 9.2 Hz, J = 3.4 Hz, H-22), 2.12-2.17 (1H, m, H-23), 2.00 - 2.12 (4H, m, H-7, 11), 1.92-1.96 (1H, m, H-23), 1.82-1.88 (1H, m, H-16<α>) 1.77 - 1.82 (2H, m, 5, 12<α>, 1), 1.70 (3H, s, 26), 1.68-1.71 (3H, m, H-6<β>, H-20, 12<β>), 1.61 - 1.68 (6H, m, H-2, H-17. H-27, H-15<β>), 1.41-1.46 (1H, m, H-16<β>), 1.28 - 1.35 (2H, m, H-1, H-6<α>), 1.18-1.24 (1H, m, H-15<α>), 1.12 (3H, s, H-28), 1.03 (3H, s, H-19), 0.94 (3H, d, J = 6.6 Hz, H-21), 0.91 (3H, s, H-30), 0.76 (3H, s, H-18)

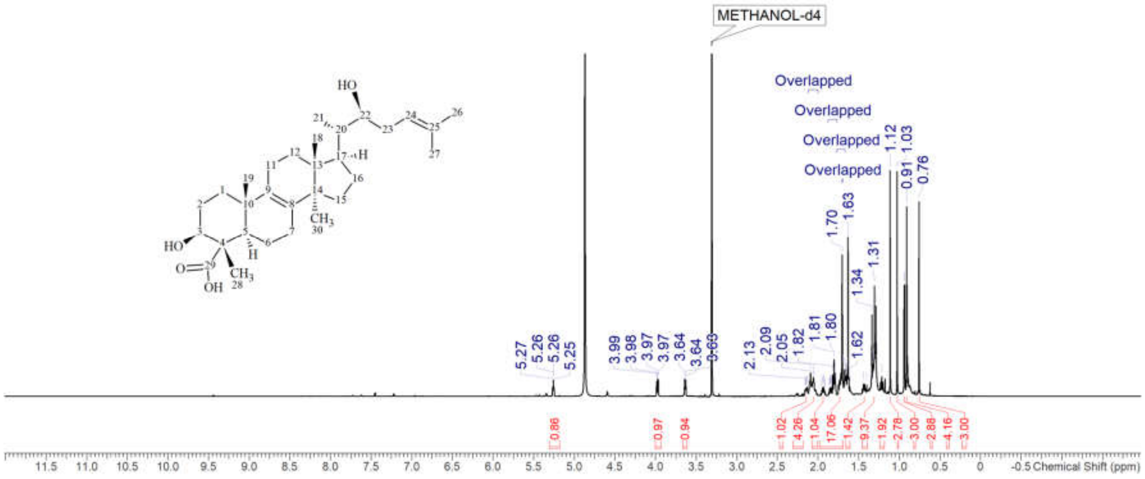

Figure S14. 800 MHz 1H NMR spectrum of compound **6**

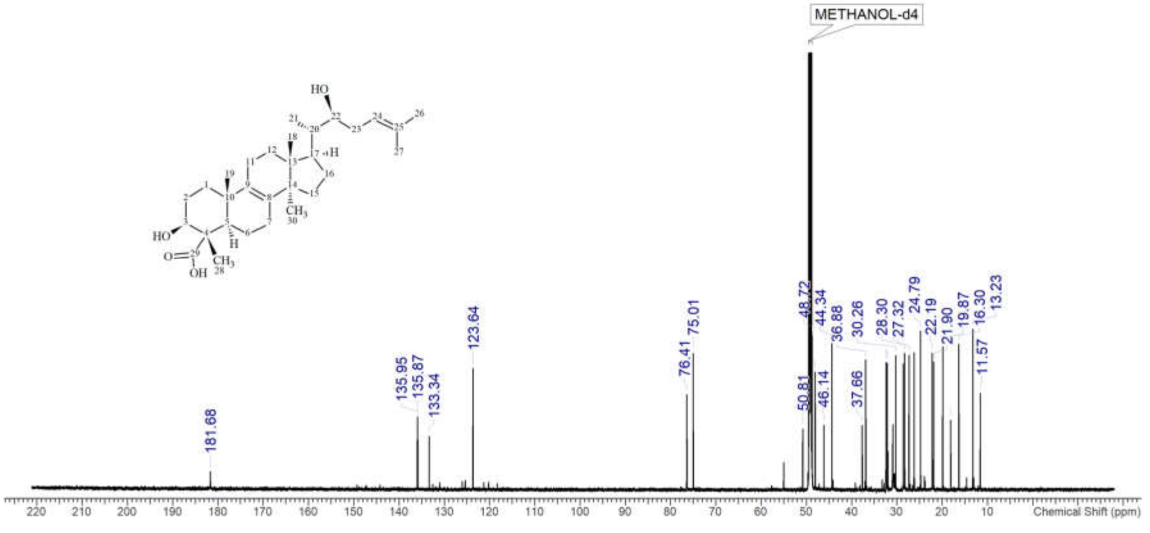

Figure S15. 200 MHz 13C NMR spectrum of compound **6**

Spectra and spectral data on compound 7

Known compound: natalic acid. ^{1}H and ^{13}C NMR assignments are in good agreement with the data available for natalic acid (some misinterpretation in the literature). [5]

^{1}H NMR (500 MHz, CHLOROFORM-d) δ = 7.38 (1H, s, H-26), 7.06 (1H, s, H-24), 3.25 (1H, dq, J = 10.4 Hz, J = 6.6 Hz, H-20), 2.46 (1H, dd, J = 12.7 Hz, J = 7.9 Hz, H-2), 2.19 - 2.27 (1H, m, H-17), 2.11 - 2.19 (4H, m, H-7, 11), 2.09 (3H, s, H-27), 2.01 - 2.09 (1H, m, H-5), 1.86 - 1.97 (1H, m, H-12), 1.78 - 1.86 (1H, m, H-16), 1.47 - 1.76 (6H, m, H-1, 2, 6, 12, 15, 1), 1.23 (3H, s, H-30), 1.20 (3H, d, J = 6.8 Hz, H-21), 1.13 - 1.19 (2H, m, H-15, 16), 0.99 (3H, s, H-19), 0.93 (3H, s, H-28), 0.82 (3H, s, H-18)

^{13}C NMR (126 MHz, CHLOROFORM-d) δ = 193.8 (C-22), 185.0 (C-29), 152.5 (C-23), 143.7 (C-26), 134.9 (C-9), 134.2 (C-8), 122.6 (C-25), 119.7 (C-24), 52.8 (C-5), 48.9 (C-14), 48.2 (C-4), 46.9 (C-17), 45.6 (C-10), 45.3 (C-13), 44.4 (C-20), 37.0 (C-2), 36.0 (C-1), 30.7 (C-15), 30.5 (C-12), 27.3 (C-16), 26.0 (C-7), 24.4 (C-28), 22.5 (C-11), 21.5 (C-30), 19.4 (C-19), 18.6 (C-6), 17.4 (C-21), 16.1 (C-18), 9.6 (C-27)

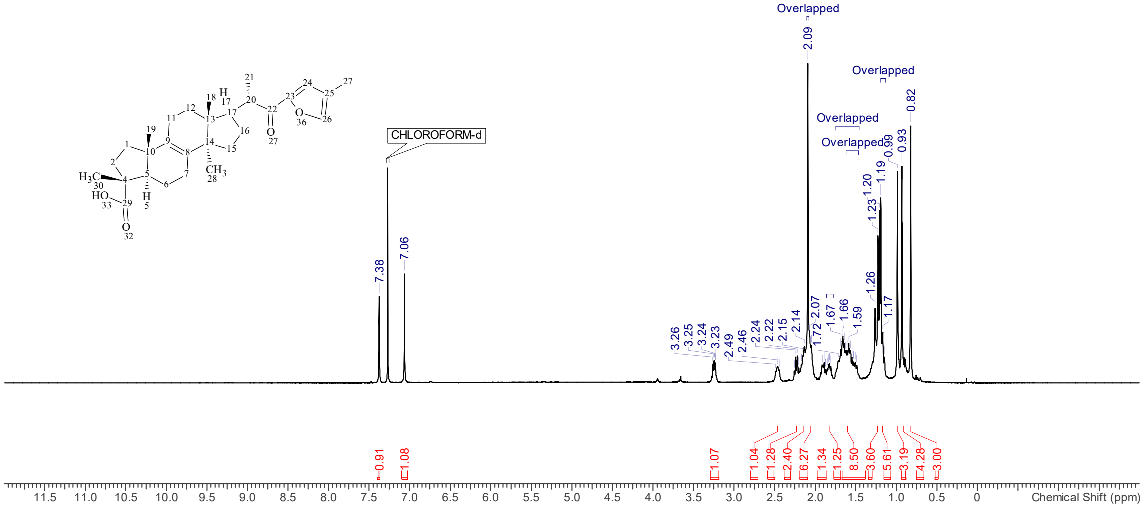

Figure S16. 500MHz 1H NMR spectrum of compound **7**

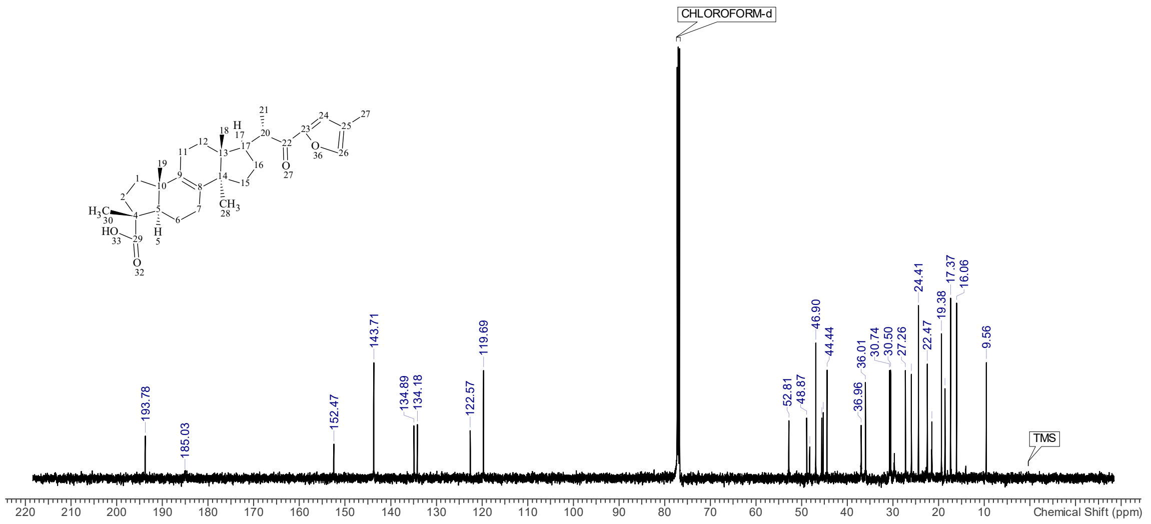

Figure S17. 125MHz 13C NMR spectrum of compound **7**

Spectra and spectral data on compound 8

Known compound, ergosta 7,22-dien-3-one (the sample contains ergosta 7-en-3-one in a ca. 3 to 1 molar ratio). The obtained NMR data are in agreement with data available in the literature.[6]

^{1}H NMR (500 MHz, CHLOROFORM-d) δ = 5.17 - 5.26 (3H, m, H-7, H-22, H-23), 2.43 (1H, td, J = 14.5 Hz, J = 5.5 Hz, H-2), 2.26 - 2.32 (1H, m, H-2), 2.23 - 2.32 (3H, m, H-2, H-4), 2.14 (1H, ddd, J = 13.3 Hz, J = 6.0 Hz, J = 2.4 Hz, H-1), 2.00 - 2.11 (2H, m, H-12, H-20), 1.80 - 1.89 (5H, m, H-5, H-6, H-24, H-14), 1.71 - 1.78 (2H, m, H-5, H-16), 1.61 - 1.67 (1H, m, H-11), 1.52 - 1.57 (1H, m, H-11), 1.37 - 1.55 (3H, m, H-1, H-15, H-25), 1.25 - 1.34 (3H, m, H-12, H-16, H-17), 1.03 (3H, d, J = 8.7 Hz, H-21), 1.03 (3H, s, H-19), 0.92 (3H, d, J = 7.4 Hz, H-28), 0.85 (3H, d, J = 6.9 Hz, H-26), 0.83 (3H, d, J = 6.9 Hz, H-27), 0.58 (3H, s, H-18)

^{13}C NMR (125 MHz, CHLOROFORM-d) δ = 211.0 (C-3), 138.5 (C-8), 134.6 (C-22), 131.0 (C-23), 116.0 (C-7), 54.9 (C-17), 54.0 (C-14), 47.8 (C-9), 43.2 (C-4), 42.3 (C-13), 41.9 (C-5), 41.8 (C-24), 39.5 (C-20), 38.3 (C-12), 37.8 (C-1), 37.1 (C-2), 33.4 (C-10), 32.1 (C-25), 29.0 (C-6), 27.1 (C-16), 21.9 (C-15), 20.7 (C-11), 20.1 (C-21), 18.9 (C-26), 18.6 (C-27), 16.6 (C-28), 11.4 (C-19), 11.1 (C-18)

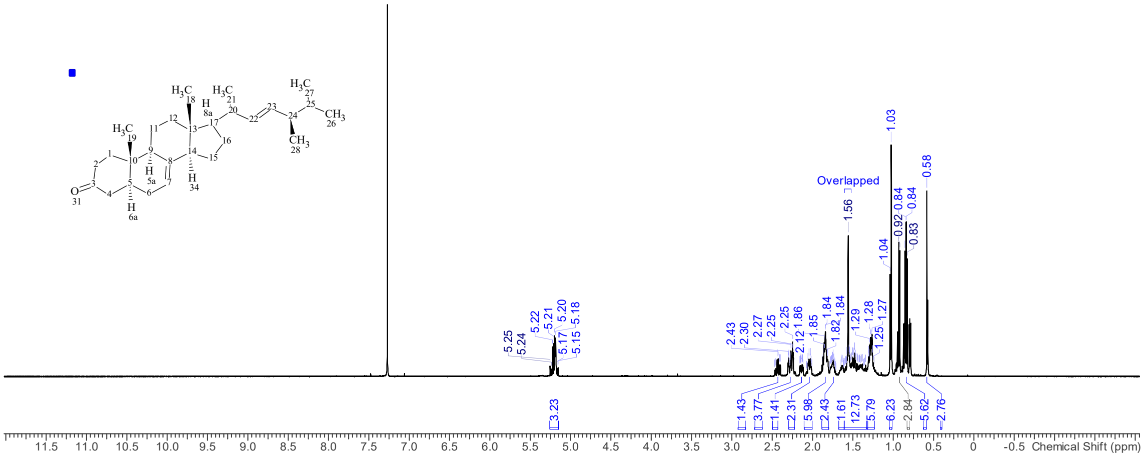

Figure S18. 500MHz 1H NMR spectrum of compound **8**

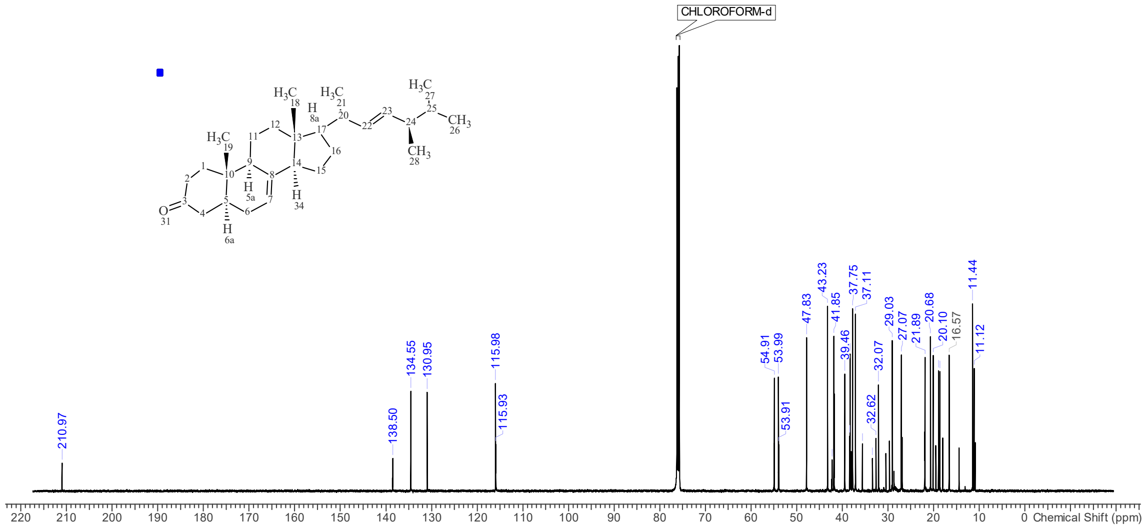

Figure S19. 125 MHz ^{13}C NMR spectrum of compound **8**

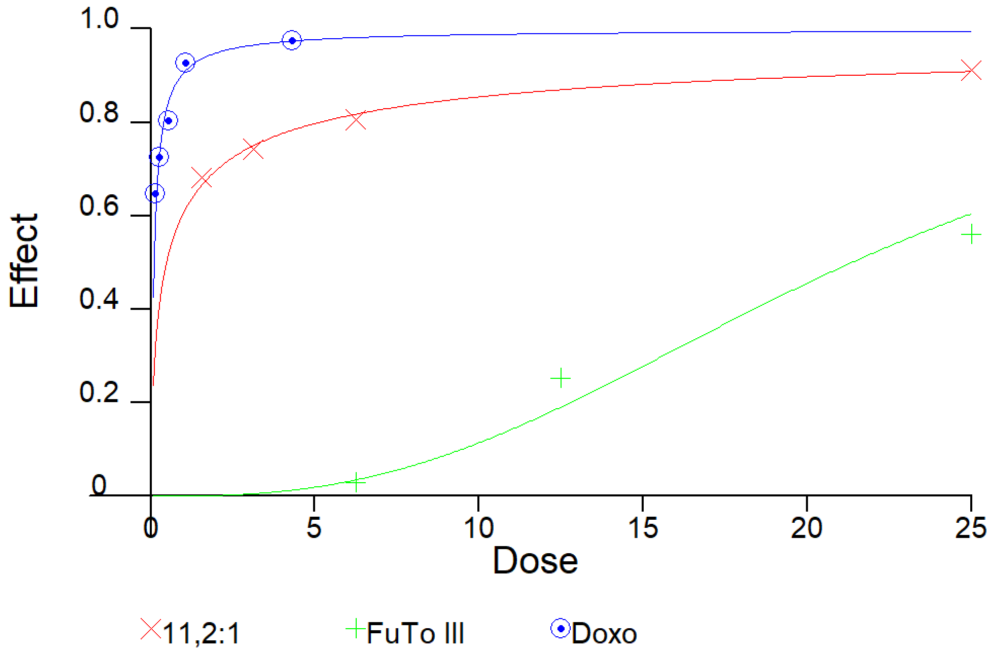

Figure S20. Cytotoxicity dose-effect curve of compound **8** (=FuTo III), doxorubicin and their combination on Colo 320 cell line

Report of the combination assay of compound **8**

Title:
Date:
Investigator:
Filename: Doxo+FuToIII_320.csn

Drug: FuTo III

Data points entered:

FuTo III uM	Fraction affected
6.25	0.0295
12.5	0.2522
25	0.5605

Median-effect parameters:

	FuTo III uM
Dm:	21.37268
lower 95%:	16.57462
upper 95%:	27.55969

m: 2.69528 +/- 0.448371
y-int: -3.5843 +/- 0.504019
r: 0.98644
CCSI: -0.9758

Drug: Doxo

Data points entered:

Doxo uM	Fraction affected
0.135	0.6475
0.269	0.7257
0.539	0.8038
1.078	0.9263
4.311	0.9749

Median-effect parameters:

	Doxo uM
Dm:	0.08504
lower 95%:	0.04717
upper 95%:	0.15331

m: 0.92513 +/- 0.087574
y-int: 0.99023 +/- 0.048874
r: 0.98682
CCSI: 0.37311

Drug: 11,2:1
Combination: FuTo III Doxo
Ratio: 11:1

Data points entered:
FuTo III Doxo Fraction affected

(uM)	(uM)		
1.56	0.141818		0.6829
3.125	0.284091		0.7419
6.25	0.568182		0.8068
25	2.27273	0.9130	

Median-effect parameters:

	FuTo III	Doxo	
	(uM)	(uM)	
Dm:	0.46705	0.04246	
lower 95%:		0.38655	0.03514
upper 95%:		0.56431	0.05130

m:	0.57876 +/- 0.040984
y-int:	0.19136 +/- 0.034715
r:	0.99502
CCSI:	-0.85056

Dose-effect table

Fa	Dose(*)	Lower 95%		Upper 95%	
0.020	0.00056	0.00201	0.00016		
0.050	0.00288	0.00825	0.00101		
0.100	0.01049	0.02514	0.00437		
0.150	0.02332	0.05013	0.01085		
0.200	0.04257	0.08431	0.02149		
0.250	0.06998	0.12958	0.03779		
0.300	0.10803	0.18868	0.06186		
0.350	0.16027	0.26552	0.09674		
0.400	0.23180	0.36570	0.14692		
0.450	0.33020	0.49734	0.21923		
0.500	0.46705	0.67268	0.32428		
0.550	0.66061	0.91072	0.47918		
0.600	0.94106	1.24255	0.71273		
0.650	1.36108	1.07561	1.72230		
0.700	2.01914	1.66203	2.45296		
0.750	3.11709	2.65692	3.65696		
0.800	5.12416	4.44406	5.90834		
0.850	9.35381	7.94430	11.01340		
0.900	20.80349		16.39366		26.39956
0.950	75.65508		50.88405		112.48499
0.990	1310.66479		601.30785		2856.84313

Note: Dose displayed is dose of first component only

CI For experimental values

FuTo III	Doxo	Fa	CI		
(uM)	(uM)				
1.56	0.141818		0.6829	0.783	
3.125	0.284091		0.741889		1.166
6.25	0.568182		0.80679	1.597	
25	2.27273	0.912987		2.595	

CI Simulations

Fa	CI	Est. s.d.	FuTo III	Doxo
			(uM)	(uM)
0.020	0.040	0.0380	0.00056	5.1002e-005
0.050	0.075	0.0573	0.00288	0.00026
0.100	0.122	0.0773	0.01049	0.00095

0.150	0.165	0.0917	0.02332	0.00212	
0.200	0.207	0.1033	0.04257	0.00387	
0.250	0.250	0.1131	0.06998	0.00636	
0.300	0.296	0.1217	0.10803	0.00982	
0.350	0.344	0.1293	0.16027	0.01457	
0.400	0.397	0.1361	0.23180	0.02107	
0.450	0.455	0.1421	0.33020	0.03002	
**0.500**	**0.521**	**0.1473**	0.46705	0.04246	
0.550	0.597	0.1517	0.66061	0.06006	
0.600	0.687	0.1554	0.94106	0.08555	
0.650	0.796	0.1584	1.36108	0.12373	
0.700	0.933	0.1613	2.01914	0.18356	
0.750	1.113	0.1665	3.11709	0.28337	
0.800	1.367	0.1819	5.12416	0.46583	
0.850	1.763	0.2335	9.35381	0.85035	
0.900	2.499	0.4057	20.80349		1.89123
0.950	4.541	1.1250	75.65508		6.87773
0.990	20.906	10.0580	1310.66479		119.15134

CI simulations with Monte Carlo simulations
200 iterations per point

Fa	CI	Mean	s.d.
0.020	0.040	0.066	0.0719
0.050	0.075	0.105	0.1006
0.100	0.122	0.153	0.1113
0.150	0.165	0.202	0.1179
0.200	0.207	0.258	0.2078
0.250	0.250	0.297	0.1365
0.300	0.296	0.312	0.1103
0.350	0.344	0.362	0.1406
0.400	0.397	0.433	0.1680
0.450	0.455	0.478	0.1535
0.500	0.521	0.566	0.1975
0.550	0.597	0.630	0.1790
0.600	0.687	0.707	0.1680
0.650	0.796	0.804	0.1848
0.700	0.933	0.958	0.1746
0.750	1.113	1.126	0.1800
0.800	1.367	1.396	0.1806
0.850	1.763	1.782	0.2363
0.900	2.499	2.542	0.4035
0.950	4.541	4.619	1.0300
0.990	20.906	23.218	10.8920

DRI for experimental values

	Drug alone			Dose Reduction Index (DRI)	
Fa	FuTo III (uM)	Doxo (uM)	FuTo III	Doxo	
0.6829	28.4098	0.1949	18.211	1.374	
0.741889		31.6215	0.2662	10.119	0.937
0.80679	36.3213	0.3987	5.811	0.702	
0.912987		51.1239	1.0793	2.045	0.475

DRI Calculations

	Drug alone			Dose Reduction Index (DRI)

Fa	FuTo III (uM)	Doxo (uM)	FuTo III	Doxo	
0.020	5.0439	0.0013	8990.497		24.836
0.050	7.1683	0.0035	2486.174		13.456
0.100	9.4584	0.0079	902.047	8.298	
0.150	11.2295	0.0130	481.531	6.152	
0.200	12.7786	0.0190	300.180	4.911	
0.250	14.2180	0.0259	203.172	4.077	
0.300	15.6075	0.0340	144.469	3.465	
0.350	16.9868	0.0436	105.991	2.989	
0.400	18.3876	0.0549	79.327	2.604	
0.450	19.8392	0.0685	60.082	2.281	
0.500	21.3727	0.0850	45.761	2.003	
0.550	23.0247	0.1056	34.854	1.759	
0.600	24.8423	0.1318	26.398	1.541	
0.650	26.8909	0.1661	19.757	1.342	
0.700	29.2675	0.2125	14.495	1.158	
0.750	32.1277	0.2789	10.307	0.984	
0.800	35.7466	0.3806	6.976	0.817	
0.850	40.6778	0.5545	4.349	0.652	
0.900	48.2948	0.9143	2.321	0.483	
0.950	63.7236	2.0506	0.842	0.298	
0.990	117.5646		12.2115	0.090	0.102

Summary table

Drug	Combination Index Values at						
	ED50	ED75	ED90	Dm	m	r	
11,2:1 (11:1)	0.52112	1.11323	2.49919	0.46705	0.57876	0.99502	
FuTo III (Not a combination)	N/A	N/A	N/A	21.37268		2.69528	0.98644
Doxo (Not a combination)	N/A	N/A	N/A	0.08504	0.92513	0.98682	

Figure S21. Histograms of modulation of P-gp efflux pump assay

Colo 205:

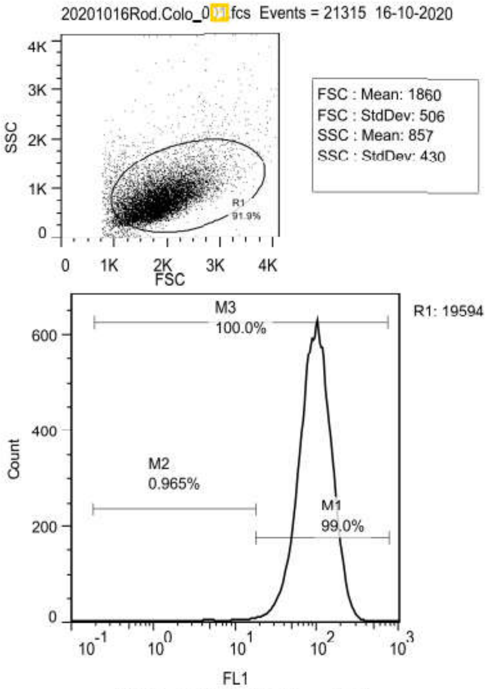

	Mean	StdDev	Count	Peak
All	101	47.3	19594	100
M1	102	46.2	19401	100
M2	8.64	5.13	189	15.4
M3	101	46.9	19590	100

Colo 320:

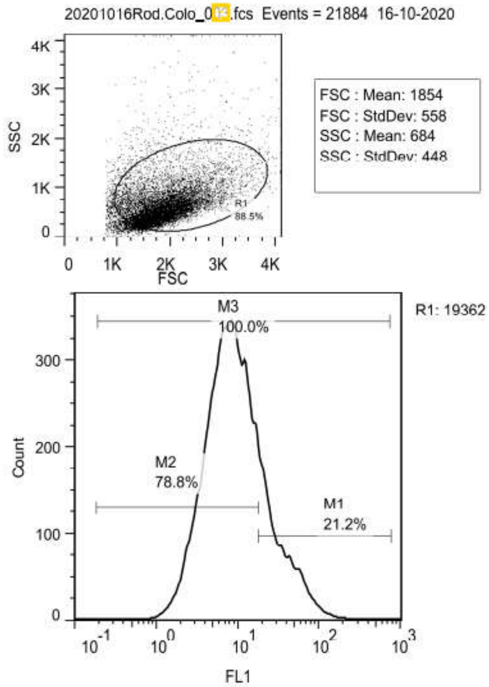

	Mean	StdDev	Count	Peak
All	14.3	17.6	19362	19.8
M1	38.2	25.9	4097	19.8
M2	7.93	4.07	15259	8.35
M3	14.3	17.6	19356	8.35

DMSO:

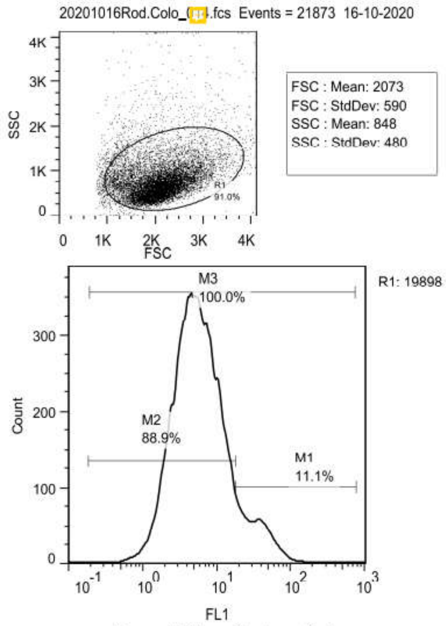

	Mean	StdDev	Count	Peak
All	9.59	13.0	19898	19.1
M1	38.3	22.3	2205	19.1
M2	6.02	3.72	17685	4.53
M3	9.59	13.0	19890	4.53

Tariquidar:

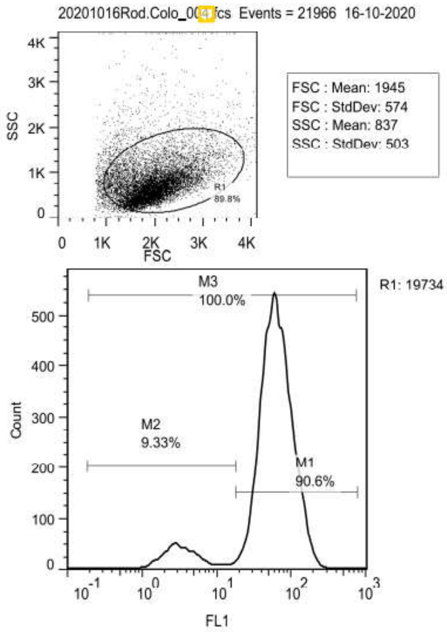

	Mean	StdDev	Count	Peak
All	64.2	39.7	19734	58.3
M1	70.3	36.5	17888	58.3
M2	4.42	3.41	1841	2.64
M3	64.1	39.4	19728	58.3

Compound **1**:

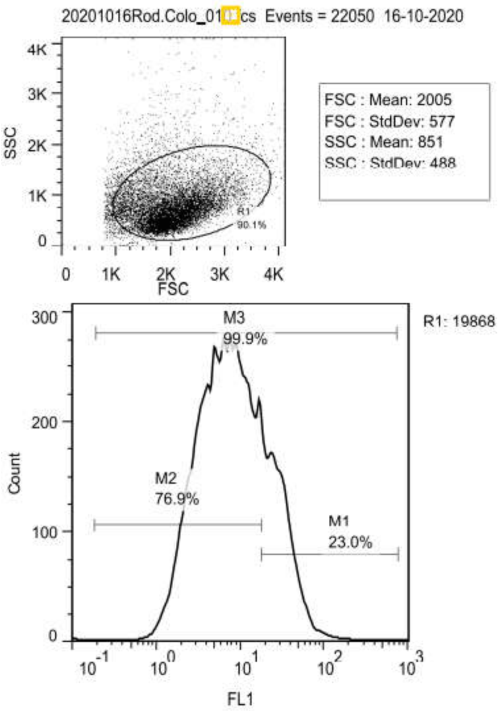

	Mean	StdDev	Count	Peak
All	13.2	15.2	19868	18.4
M1	33.5	20.1	4575	18.4
M2	7.08	4.30	15283	4.87
M3	13.2	15.2	19858	4.87

Compound **6**:

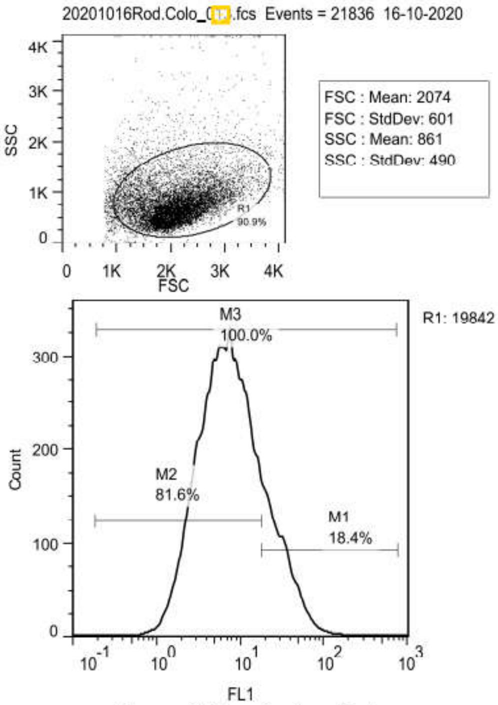

	Mean	StdDev	Count	Peak
All	11.9	14.0	19842	20.5
M1	33.9	20.3	3642	20.5
M2	7.01	4.09	16197	7.23
M3	11.9	14.0	19839	7.23

Compound **7**:

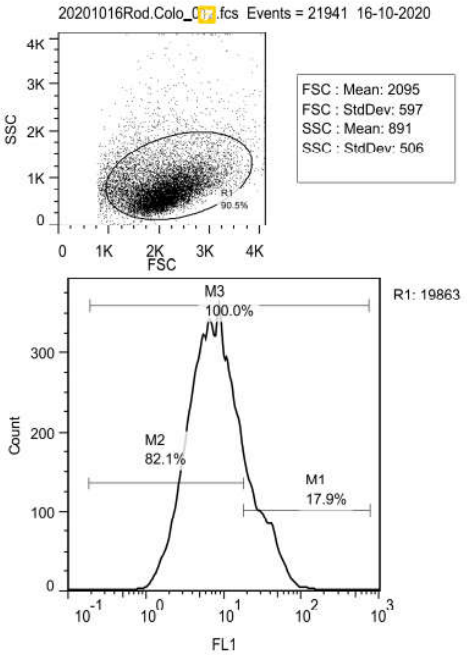

	Mean	StdDev	Count	Peak
All	12.2	14.3	19863	19.1
M1	34.1	21.7	3559	19.1
M2	7.38	4.03	16300	8.66
M3	12.2	14.3	19859	8.66

Compound **8**:

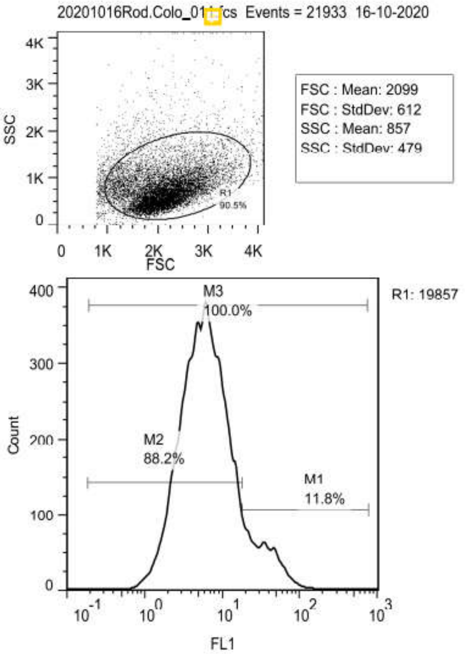

	Mean	StdDev	Count	Peak
All	10.1	13.3	19857	18.4
M1	38.1	22.5	2342	18.4
M2	6.41	3.76	17508	6.26
M3	10.1	13.3	19850	6.26

References

1. Kim, J.-P.; Yun, B.-S.; Shim, Y.K.; Yoo, I.-D. Inoscavin A, a new free radical scavenger from the mushroom *Inonotus xeranticus*. *Tetrahedron Lett*. **1999**, *40*, 6643–6644.
2. Ashok K. Batta & Srinivasa Rangaswami. Crystalline chemical components of *Fomes senex* and structure of senexdiolic acid and related compounds. *J. Chem. Soc. Perkin Trans. 1 Org. Bio-Org. Chem.* **1975**, 451–455.
3. González, A.G.; Expósito, T.S.; Barrera, J.B.; Castellano, A.G.; Marante, F.J.T. The absolute stereochemistry of senexdiolic acid at C-22. *J. Nat. Prod.* **1993**, *56*, 2170–2174.
4. Ashok K. Batta & Srinivasa Rangaswami. New tetracyclic triterpenes from *Fomes senex*: senexonol, senexdione, oxidosenexone, and senexdiolic acid. *Curr. Sci.* **1970**, *39*, 416–417.
5. González, A.G.; Expósito, T.S.; Toledo Marante, F.J.; Pérez, M.J.M.; Tejera, E.B.; Bermejo Barrera, J. Lanosterol derivatives from *Phellinus torulosus*. *Phytochemistry* **1994**, *35*, 1523–1526.
6. Jain, A.C.; Gupta, S.K. The isolation of lanosta-7,9(11),24-trien-3β,21-diol from the fungus *Ganoderma australe*. *Phytochemistry* **1984**, *23*, 686–687.
